# Global patterns in soil seed bank seasonality: a meta-analysis of ecosystem and functional group dynamics

**DOI:** 10.3389/fpls.2025.1725507

**Published:** 2025-12-17

**Authors:** Li Peng, Yafei Shi, Dong Lin, Furong Niu, Xiaoni Liu, Yuzhen Wang

**Affiliations:** 1 Pratacultural College, Gansu Agricultural University, Lanzhou, China; 2 College of Forestry, Gansu Agricultural University, Lanzhou, China

**Keywords:** seed bank, transient seed bank, persistent seed bank, seasonal variation, meta-analysis

## Abstract

**Introduction:**

Soil seed banks are key components driving vegetation dynamics and maintaining ecosystem resilience, and their composition and abundance play crucial roles in the establishment, maintenance, and regeneration of plant communities. Although previous studies have documented seasonal variation in soil seed banks, most have been limited to local or regional scales.

**Methods:**

To address this gap, we conducted a global meta-analysis based on 1,018 paired observations from diverse regions to systematically assess changes in soil seed bank density and species richness across early, mid, and late growing seasons.

**Results:**

Our results show that soil seed banks exhibit seasonal variation at the global scale, with patterns differing among ecosystem types and plant functional groups, and some differences reaching statistical significance. Specifically, annuals and legumes had higher seed bank densities in the late season compared to the mid-season. Grassland ecosystems showed the most pronounced seasonal fluctuations, with mid-season densities significantly lower than in the early and late growing seasons. In addition, transient seed banks supported significantly higher species richness than persistent seed banks.

**Conclusion:**

These findings reveal the seasonal dynamics of soil seed banks at the global scale and demonstrate how ecosystem types and plant functional groups influence these patterns. Overall, this study provides new insights into plant community succession and ecosystem management.

## 1 Introduction

Soil seed banks form a vital foundation for plant regeneration and community recovery, being regarded as a potential “latent vegetation layer” (Leck et al., 1989; Wang et al., 2011). Extensive research indicates that soil seed banks play a pivotal role in maintaining species diversity, enhancing ecosystem stability, and supporting the recovery of degraded ecosystems (Wambugu et al., 2023; Guo et al., 2023). However, existing studies predominantly focus on seed bank size, species composition, and their relationship with aboveground vegetation (Vandvik et al., 2016; Lu et al., 2020), with limited systematic quantification of the intras seasonal dynamics of soil seed banks.

Seed banks are constantly shaped by the dynamic interplay of seed input, germination, mortality, predation, and dormancy transitions (Goodson et al., 2001). At the same time, aboveground vegetation follows distinct seasonal rhythms. Its phenological processes—growth, fruiting, and senescence—govern not only seed input but also influence seed survival and germination through bio-environmental interactions (Chalermsri et al., 2020; Yang et al., 2023; Figueroa et al., 2004). It can thus be inferred that seed banks are neither homogeneous nor static, but exhibit periodic fluctuations that correspond to the dynamics of the aboveground plant community.

In seed bank research, the distinction between transient and persistent seed banks has long relied on seasonal sampling. Transient seed banks are generally defined as those in which seeds persist in the soil for less than one year, whereas persistent seed banks are those in which seeds remain viable in the soil for more than one year (Thompson and Grime, 1979; Bakker et al., 1996; Walck et al., 2005). This classification framework provides a foundation for understanding seed persistence strategies and their ecological significance, including the role of soil seed banks in promoting ecosystem resilience (Yan et al., 2005). By distinguishing between transient and persistent seed banks, researchers can examine seed bank species composition, seed density, and their relationships with aboveground vegetation, thereby gaining insights into potential community dynamics and future successional trajectories (Wang et al., 2024). Although this framework has been widely applied, most studies have focused on static comparisons, primarily investigating the relationships between seed bank types and aboveground vegetation, while systematic analyses of seasonal dynamics in seed bank density and species composition throughout the growing season remain limited.

The seasonal dynamics of soil seed banks provide essential insights into vegetation formation and renewal. They also determine how seed banks function across ecosystems and plant functional groups, where responses to temporal variation can differ substantially. Such differences, in turn, form a crucial basis for distinguishing between transient and persistent seed banks (Ma et al., 2013a). Based on this, we analyzed 1,018 paired data records extracted from global peer-reviewed literature to quantify the intra-annual dynamics of soil seed banks worldwide, systematically revealing their seasonal variation patterns. We hypothesize that the composition and density of soil seed banks exhibit regular seasonal variations similar to those of aboveground vegetation, and that these dynamics are further reflected in seasonal shifts between transient and persistent seed banks. To test this hypothesis, we constructed a global soil seed bank database from existing public data and conducted a meta-analysis to examine: (1) changes in seed richness and density across early-, mid-, and late-growing seasons; (2) seasonal shifts in the relative contributions of transient and persistent seed banks; (3) differences among plant functional types across these phases; and (4) variations in seed bank dynamics across ecosystem types.

## 2 Materials and methods

### 2.1 Data collection

In December 2024, we conducted a literature search of peer-reviewed articles from 1975 to the present using the Web of Science Core Collection and the China National Knowledge Infrastructure (CNKI) database. The literature search was conducted using Boolean operators to combine key terms. The search syntax included: TS= (“seed bank”) AND TS= (season* OR temporal OR dynamic* OR “within-year” OR “intra-annual” OR “growing season”).

We screened a total of 2,721 relevant publications and further filtered and extracted data according to explicit criteria. These were then rigorously screened to extract studies containing original soil seed bank composition data and specific sampling time information, ensuring the comparability and representativeness of the data used in the analysis. The inclusion criteria were defined as follows: (1) Studies must include at least two independent soil seed bank samplings at different stages of the plant growing season (e.g., early, middle, and late stages). Sampling spanning natural years is also acceptable. (2) Studies must report at least one soil seed bank parameter, such as seed density or species richness. Exclusion criteria include: (1) studies whose timeframe does not cover the growing season; (2) studies with sampling intervals too short (e.g., 1–2 months) to capture meaningful temporal variation, unless the study explicitly examines seasonal changes.

Following the PRISMA guidelines (**Supplementary Figure S1**), 109 studies from 1975 to 2024 meeting the inclusion criteria were selected. Soil seed bank data were systematically extracted based on predefined screening criteria. We also collected geographic and climatic variables for each site—including latitude, longitude, elevation, mean annual temperature (MAT), and mean annual precipitation (MAP). Missing environmental data were supplemented using the WorldClim database based on geographic coordinates. Key seed bank parameters such as seed density and species richness were directly taken when available in tables, or digitized from figures using WebPlotDigitizer (v.4.8) (Burda et al., 2017).

**Figure 1:**
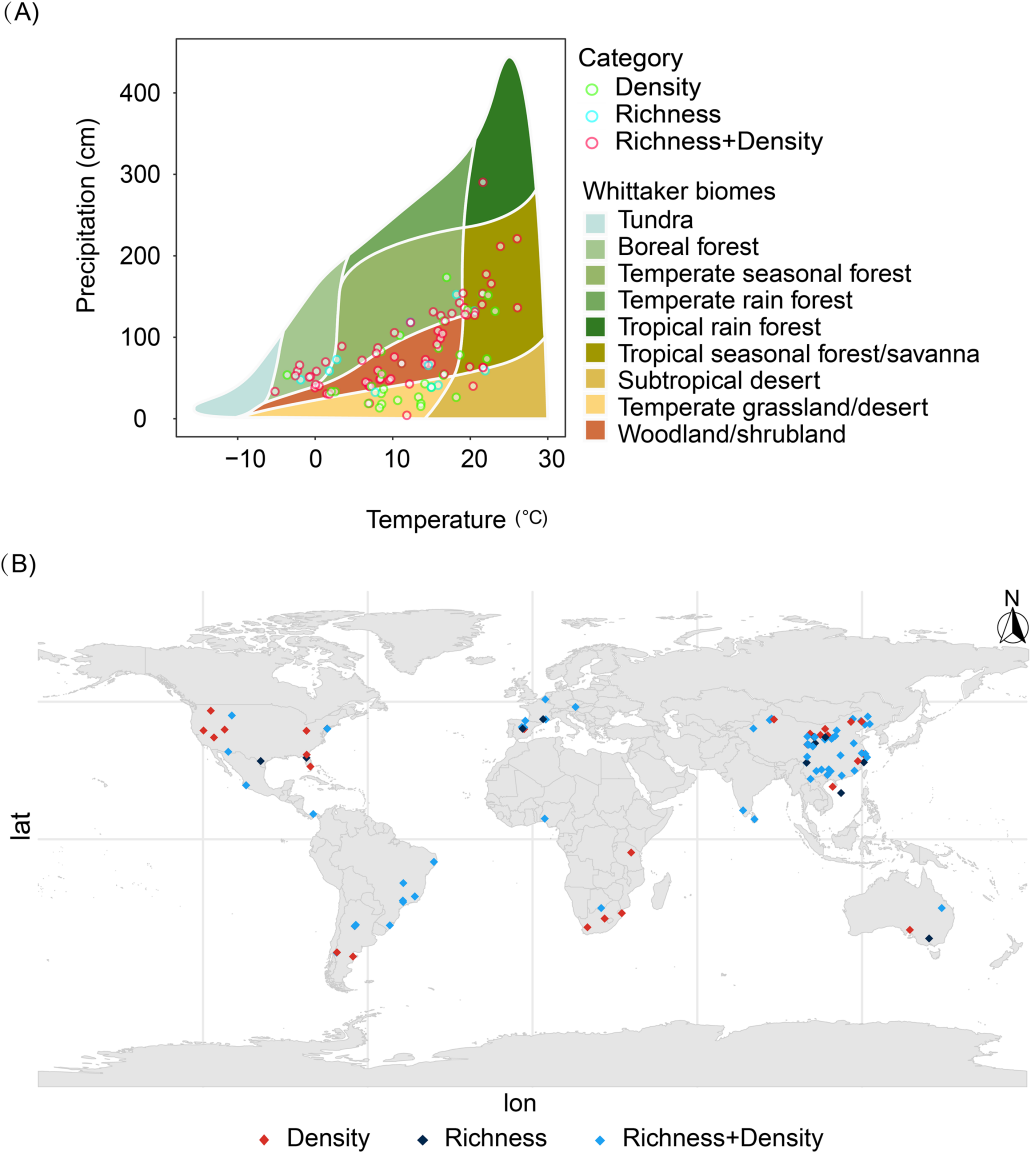
Geographic distribution of survey sites covered in this study **(A)** and biogeographic regions **(B)**. Green hollow circles indicate sites providing only seed bank density data, blue hollow circles indicate sites providing only species richness data, and red hollow circles indicate sites providing both density and richness data.

A total of 1,018 paired data points were compiled and analyzed from 109 publications, constituting a global dataset that includes multiple countries and regions (**Figure 1A**). Based on these standardized ecosystem classifications, we compared soil seed bank density and species richness across different growing season stages within each ecosystem type to elucidate the spatiotemporal patterns of seed bank dynamics (Stibig et al., 2007). Ecosystem types are regarded as critical environmental variables for studying spatial variations in global-scale soil seed bank characteristics. Based on descriptions from original research sources, an ecosystem classification system was systematically compiled and standardized. To address inconsistencies in ecosystem nomenclature, a unified approach was achieved by integrating geographic location, vegetation type, and research context information, ensuring comparability across studies. Subsequently, each study site was positioned within the climatic space according to the Whitaker biome classification system to determine its biome type (**Figure 1B**).

### 2.2 Definition method of soil seed bank stages

This study divides the soil seed bank into three phases: early-growing season, mid-growing season, and late-growing season. This classification method follows Thompson and Grime (1979), inferring the corresponding growing season stage of the seed bank based on the relative sampling time of seed production within the phenological period. To enhance classification accuracy, this study further utilizes remote sensing phenological information from the MODIS MCD12Q2 global land surface phenology dataset (Sulla-Menashe et al., 2019) to validate and assist in determining the start and end dates for each growing season stage.

In tropical regions, where temperature variations are minimal and seasonal patterns are often indistinct, the traditional temperate “growing season” framework cannot be directly applied. To address this challenge, we established a unified classification standard: based on long-term monthly precipitation data, the three consecutive months with the highest cumulative rainfall are defined as the mid-growing season. The preceding three months are designated as the early-growing season, and the following three months as the late-growing season. When original literature provided explicit sampling dates or phenological details, we prioritized this information; otherwise, classification was based on climate data. Considering tropical samples constitute only about 10% of the dataset, this classification method has minimal impact on the overall logic and robustness of the analysis results.

### 2.3 Definition of transient and persistent soil seed banks based on sampling seasons

Researchers typically classify seed banks as transient or persistent based on sampling timing, determining their type by observing seed retention at different time points. Comparing the two reveals seasonal variations in seed banks and quantifies the contribution of transient seeds to community maintenance. Based on this approach, and to systematically integrate data from diverse studies, this research categorizes seed banks into three types according to original literature classifications: Transient Seed Bank Type II (T+P-II), typically sampled in the early-growing season, composed of short-lived seeds poised to germinate after overwintering; Persistent seed banks (P), typically sampled in the mid-growing season, comprised of accumulated long-dormant seeds; Transient seed banks I (T+P-I), typically sampled in the late-growing season, comprised of newly dispersed short-lived seeds.

### 2.4 The method for defining functional groups

To understand functional composition differences in soil seed banks across different stages of the growing season, this study hypothesizes that seed banks at various stages may exhibit not only overall differences in species richness and density but also specific variations in functional group composition. To test this hypothesis, we reviewed original research literature and extracted species information where data were available. Based on these trait data, species were classified into three functional groups: (1) Life history type—annuals and perennials; (2) Plant form—herbaceous, shrubby, arboreal, and lianas; (3) Major plant groups—Poaceae (grasses), Cyperaceae (sedge-like plants), Fabaceae (legumes), and other weed-like herbaceous groups. Subsequently, using this functional group classification system, we analyzed changes in the functional composition of soil seed banks across different stages of the growing season.

### 2.5 Ecosystem type classification

To clarify the ecological context of study sites, this research defined the ecosystem types to which each site belonged based on existing literature and the Global Ecosystem Classification System. Primary categories included forests, grasslands, shrublands, and farmlands. Defining ecosystem types facilitates understanding the structural characteristics and functional composition differences of soil seed banks under varying environmental conditions. Building upon this foundation, we analyzed the composition and quantitative changes in to clarify the ecological context of study sites, this research defined the ecosystem types to which each site belonged based on existing literature and the Global Ecosystem Classification System. Primary categories included forests, grasslands, shrublands, and farmlands. Defining ecosystem types facilitates understanding the structural characteristics and functional composition differences of soil seed banks under varying environmental conditions. Building upon this foundation, we analyzed the composition and quantitative changes in seed banks across different growth stages within each ecosystem. This enabled us to compare seasonal variations between ecosystems and explore the potential impacts of different environmental contexts on seed bank dynamics.

### 2.6 Statistical analysis

To quantify the dynamic changes in soil seed banks across different stages, this study employs the natural logarithm of the response ratio (lnRR) as the effect size, calculated as follows:

(1) $$\ln RR=\ln\left({\bar{x}}_{t}∕{\bar{x}}_{c}\right)$$

Where ${\bar{x}}_{t}$ and ${\bar{x}}_{c}$ represent the average seed bank density or species richness at two sampling times (for example, early-season and mid-season), respectively (Wasserman et al., 1988). Each data record includes at least one comparison between two stages, reflecting the temporal changes in the seed bank.

The effect size was converted into effect size (%) to facilitate intuitive interpretation of the temporal dynamics of the seed bank.

(2) Effect size (%)=(exp (lnRR++)−1)×100%

where E > 0 if ${\bar{x}}_{t}>{\bar{x}}_{c}$, while E< 0 if ${\bar{x}}_{t}<{\bar{x}}_{c}$.

Meta-analyses were conducted using random-effects models, with study ID included as a random factor to account for variation both between and within studies. When studies reported multiple sets of observations, the random factor controlled for the nested structure of the data. Effect sizes were estimated using mixed-effects models with parameters obtained via restricted maximum likelihood (REML).

With only 15% of included studies reporting standard deviations or standard errors, the number of replicates per study was adopted as the primary weighting factor (Xu et al., 2020; Oliveira et al., 2020; Huang et al., 2023). To ensure robustness of the results, additional analyses were performed. The main analyses were repeated using the subset of studies reporting variance estimates and using equal weighting schemes (Doncaster et al., 2018). Sensitivity analyses included a leave-one-out (LOO) analysis for the overall effect, which confirmed that the conclusions remained consistent across different weighting approaches (see **Supplementary Figures S4**-**S6**).

Heterogeneity was assessed for all main comparisons. Variance components (τ²) were calculated, and I² was used to estimate the proportion of variance attributable to true differences (Nakagawa and Santos, 2012). Cochran’s Q statistic was also calculated as a classical measure of heterogeneity. Detailed τ², I², and Q values are provided in **Supplementary Tables S1**-**S5**.

To assess publication bias, Egger’s regression test for asymmetry was applied to compare seed density and abundance across three time periods (Duval and Tweedie, 2000; Peters et al., 2008). Where sample sizes permitted, comparisons showing significant funnel plot asymmetry (*P* < 0.05) were corrected using the Trim and Fill method. The corrected results indicated robust overall effects (see **Supplementary Figures S1**, **S3**), suggesting that publication bias is unlikely to significantly influence the conclusions of this study. All analyses were performed in R using the metafor package (Viechtbauer, 2010).

## 3 Results

### 3.1 Comparison of soil seed bank richness and density among early, mid, and late growth stages

**Figure 2:**
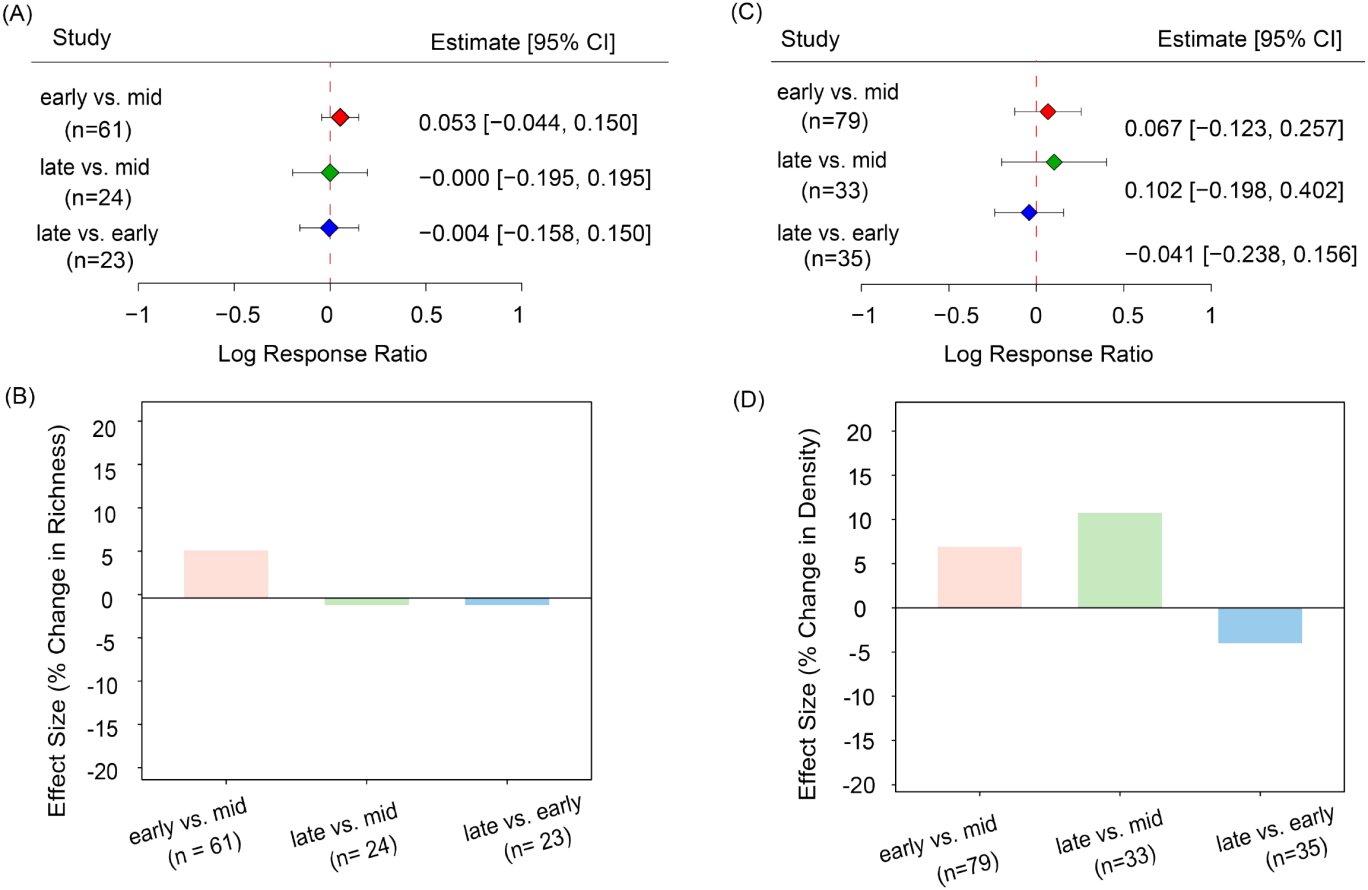
Pairwise effect sizes (log response ratios) for soil seed bank species richness and seed density between growing-season stages. **(A)** Effect sizes for species richness; **(B)** percentage change in species richness derived from pairwise effect sizes; **(C)** effect sizes for seed density; **(D)** percentage change in seed density derived from pairwise effect sizes. Each point represents the estimated effect size (Log Response Ratio), with error bars indicating the 95% confidence interval (CI). Colors distinguish different comparison groups: red for early-growing season vs. mid-growing season, green for late-growing season vs. mid-growing season, and blue for late-growing season vs. early-growing season.

Based on Equations (1) and (2), species richness in the early growing season was approximately 5.4% higher than in the mid-growing season [effect size (hereafter referred to as es)] 0.053, confidence interval (hereafter referred to as CI) -0.044 to 0.150, **Figures 2A, B**). Similarly, seed density was 6.9% greater in the early growing season compared to the mid-growing period (es = 0.067, CI = -0.123 to 0.25). Seed density in the late-growing season was also about 10.7% higher than in the mid-growing season (es = -0.102, CI = -0.198 to 0.402, **Figures 2C, D**). Notably, seed density exhibited an overall dynamic trend, characterized by being high in the early growing season, low in the mid-growing season, and rebounding in the late growing season. Overall, this pattern indicates potential seasonal fluctuations in seed banks throughout the growing season, suggesting that these seed bank dynamics may be influenced by seasonal factors, albeit with relatively moderate variations in their overall size and composition.

### 3.2 Comparative relationship of richness and density between transient and persistent seed banks

**Figure 3:**
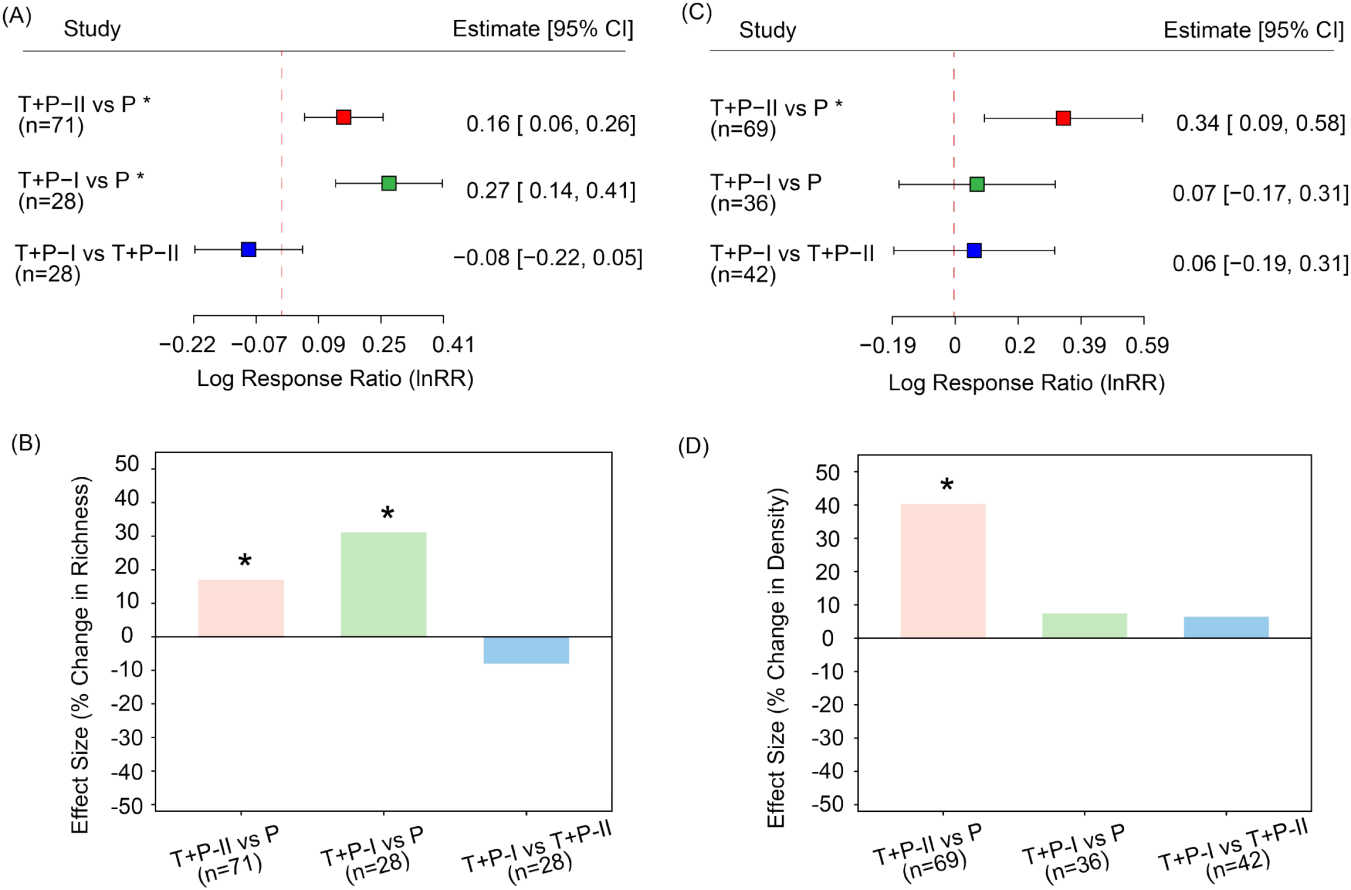
Pairwise comparisons of seed bank types. Panel **(A)** shows species richness for each pairwise comparison, and panel **(B)** shows the corresponding percentage change in species richness. Panel **(C)** shows seed density for each pairwise comparison, and panel **(D)** shows the percentage change in seed density. Asterisks (*) indicate statistically significant differences between the two compared stages (P< 0.05), and *n* represents the number of pairwise comparisons included. Based on the classification in the original literature, soil seed banks can be categorized into three types according to the sequence of the growing season: early-growing season (transient type II, T+P-II); mid-growing season (persistent type, P); and late-growing season (transient type I, T+P-I).

Based on the results of Equations (1) and (2), results based on pairwise effect sizes indicate that temporary seed banks have higher species richness than permanent seed bank. Specifically, Temporary Type I seed banks had higher species richness than Permanent seed banks (es = 0.27, CI = 0.14 to 0.41, **Figure 3A**), corresponding to an approximately 31.1% increase (**Figure 3B**). Similarly, Temporary Type II seed banks had higher species richness than Permanent seed banks (es = 0.16, CI = 0.06 to 0.26), with an increase of about 17.0% (**Figures 3A, B**). In terms of seed density, the interim Category II seed vault also exhibited a significantly higher value than the permanent repository (es = 0.34, 95% CI = 0.09 to 0.58), representing an increase of approximately 40.3% (**Figures 3C, D**). Interestingly, seed density exhibited considerably greater variability than species richness, indicating that seed size are more sensitive to seasonal succession and environmental fluctuations than species composition.

### 3.3 Functional group composition of soil seed banks at different stages

**Figure 4:**
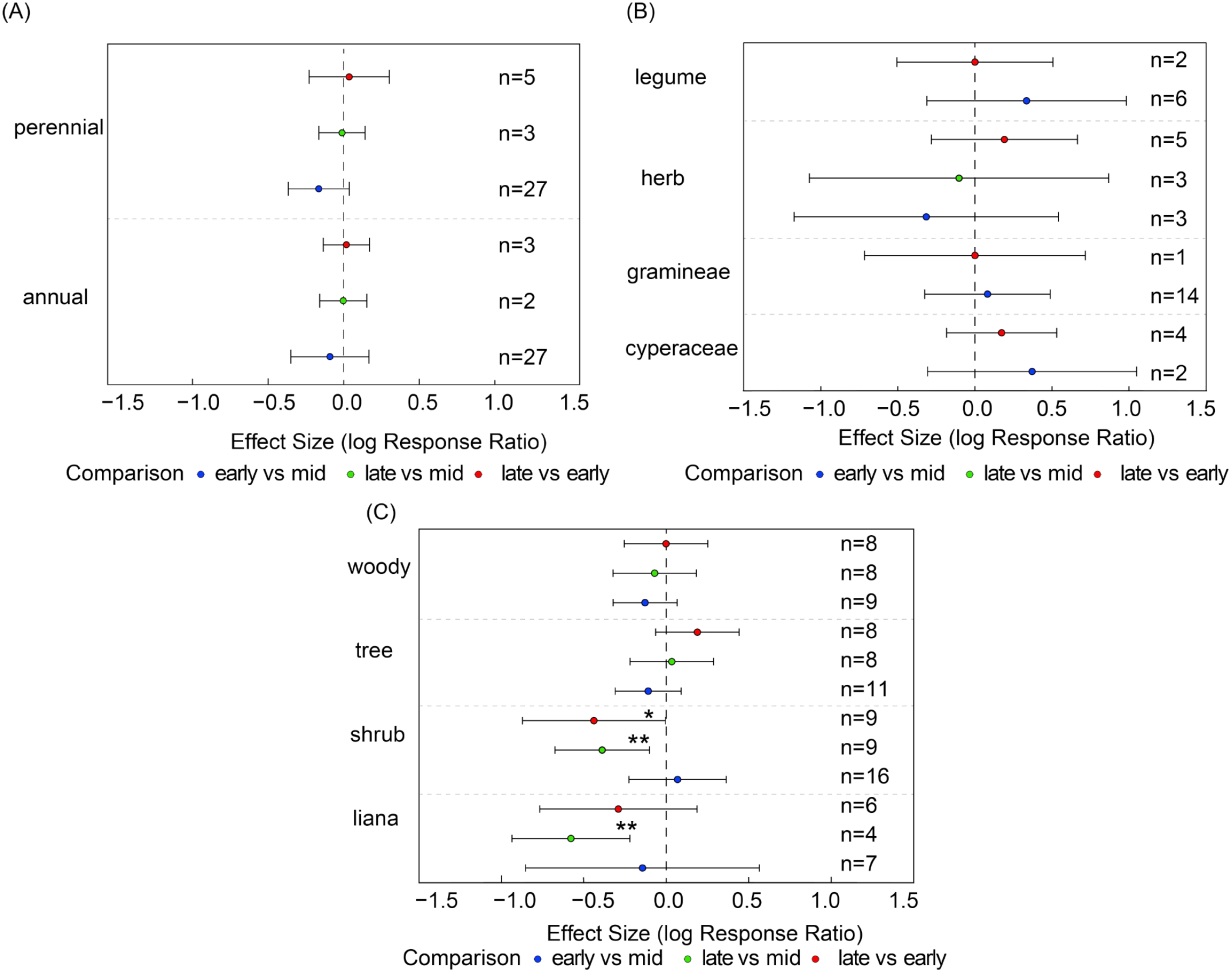
For each functional group, species richness was compared pairwise between growth stages. Panels show species richness of **(A)** life-history types (annuals, perennials), **(B)** major plant groups (Gramineae, Cyperaceae, Leguminosae, other herbs), and **(C)** growth forms (shrubs, trees, woody species, liana). Points show effect estimates with 95% confidence intervals. Blue indicates comparisons between early- and mid-growing seasons, orange indicates comparisons between late- and mid-growing seasons, and green indicates comparisons between late- and early-growing seasons. Asterisks denote significance levels (*P < 0.05, **P < 0.01, and *n* represents the number of samples.

As shown in Equation (1), the seed bank species richness of most plant functional groups did not exhibit significant changes across different growth stages, indicating relatively stable community structures (**Figures 4A, B**). However, certain functional groups exhibited high sensitivity to seasonal variation: liana species richness was significantly lower in the late growing season compared to the mid-season (es = -0.58, 95% CI: -0.94 to -0.22, **Figure 4C**); shrub species richness was significantly lower in the late season compared to both the early (es = -0.44, 95% CI= -0.87 to -0.01) and mid-season (es = -0.39, 95% C= -0.68 to -0.10, **Figure 4C**).

In contrast, no significant differences were observed across different growth stages for other functional groups such as herbaceous plants and trees, nor for life history types like annuals and perennials (**Figures 4A–C**). Although the overall community structure remained relatively stable, the dynamic changes in certain functional groups revealed their heightened sensitivity to seasonal environmental factors.

**Figure 5:**
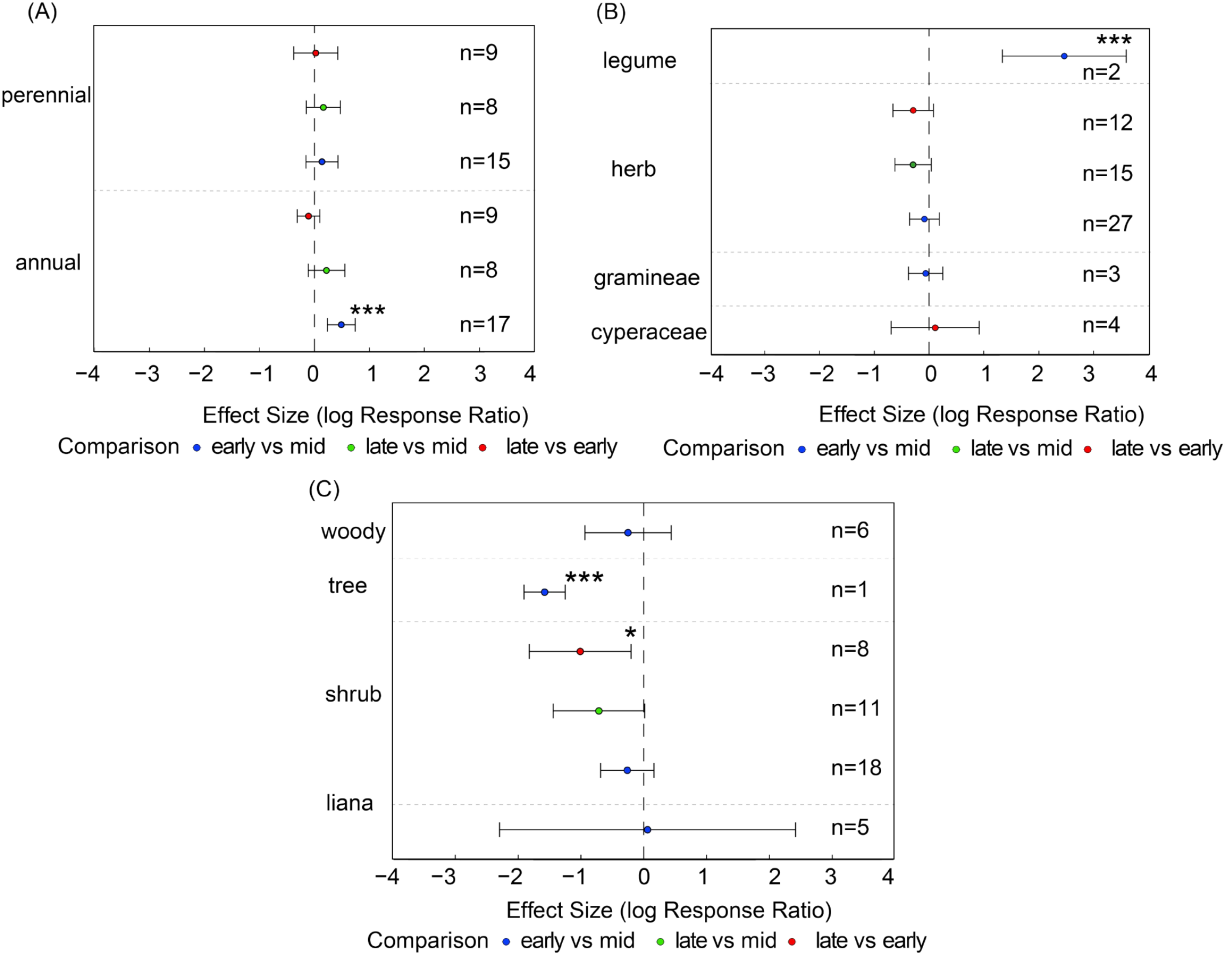
Seed density of plant functional groups based on pairwise comparisons between different growing-season stages. Panels show seed density of **(A)** life-history types (annuals, perennials), **(B)** major plant groups (Gramineae, Cyperaceae, Leguminosae, other herbs), and **(C)** growth forms (shrubs, trees, woody species, lianas). Points show effect estimates with 95% confidence intervals. Blue indicates comparisons between early- and mid-growing seasons, orange indicates comparisons between late- and mid-growing seasons, and green indicates comparisons between late- and early-growing seasons. Asterisks denote significance levels (*P < 0.05, ***P < 0.001), and *n* represents the number of samples.

Compared with the mid-growing season, annual plants exhibited significantly higher seed bank densities in the early-growing season (es = 0.49, CI = 0.23 to 0.74, **Figure 5A**). Similarly, legume seed density in the early-growing season was significantly higher than in the mid-growing season (es = 2.47, CI = 1.34 to 3.60, **Figure 5B**), indicating that functionally dominant groups can rapidly exploit early-season resources to establish population dominance. In contrast, shrub seed density in the late-growing season was significantly lower than in the early-growing season (es = -1.01, CI = -1.8 to -0.20), whereas tree seed density in the early-growing season was significantly lower than in the mid-growing season (es = -1.58, CI = -1.91 to -1.25, **Figure 5C**). Other functional groups showed no significant differences across growth stages, indicating relatively stable population dynamics.

### 3.4 Seasonal variation of soil seed banks across different ecosystem types

**Figure 6:**
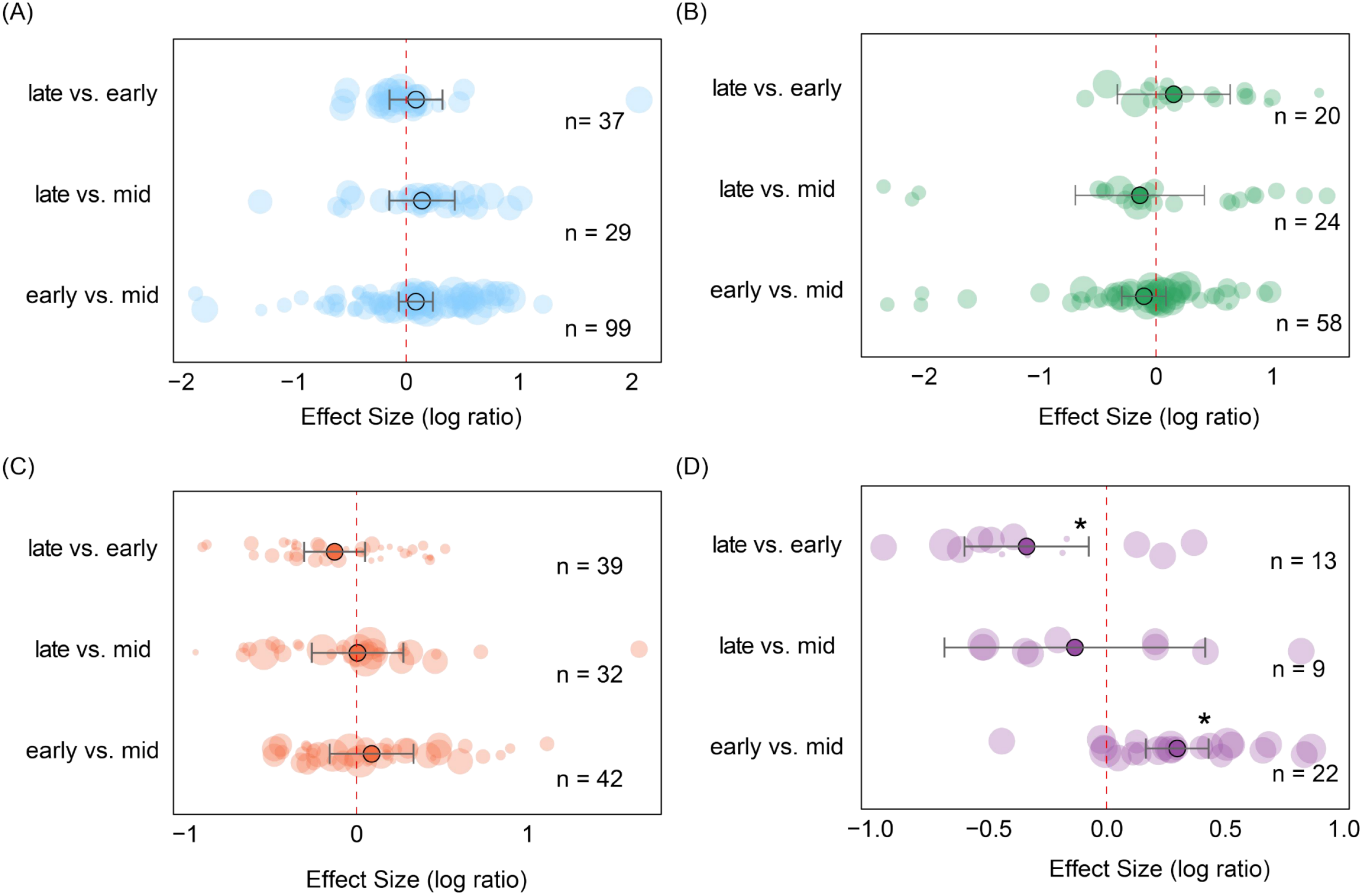
Changes in soil seed bank species richness across ecosystems based on pairwise comparisons between growing-season stages. **(A)** grassland; **(B)** forest; **(C)** shrubland; **(D)** farmland. Each point represents the effect size between two growing-season stages. Error bars denote 95% confidence intervals. Circle size corresponds to sample size in the respective comparison, with larger circles indicating greater sample numbers, and *n* represents the number of samples. Asterisks indicate statistical significance levels (*P < 0.05,).

According to Equation (1), in natural ecosystems such as grasslands, forests, and shrublands, the species richness of soil seed banks fluctuates slightly throughout the growing season (**Figures 6A–C**) but remains generally stable. In contrast, farmland ecosystems (**Figure 6D**) exhibit more pronounced seasonal variations. Species richness fluctuates significantly throughout the growing season, potentially linked to agricultural management disturbances, sowing and harvesting schedules, and the rapid growth of short-season crops.

**Figure 7:**
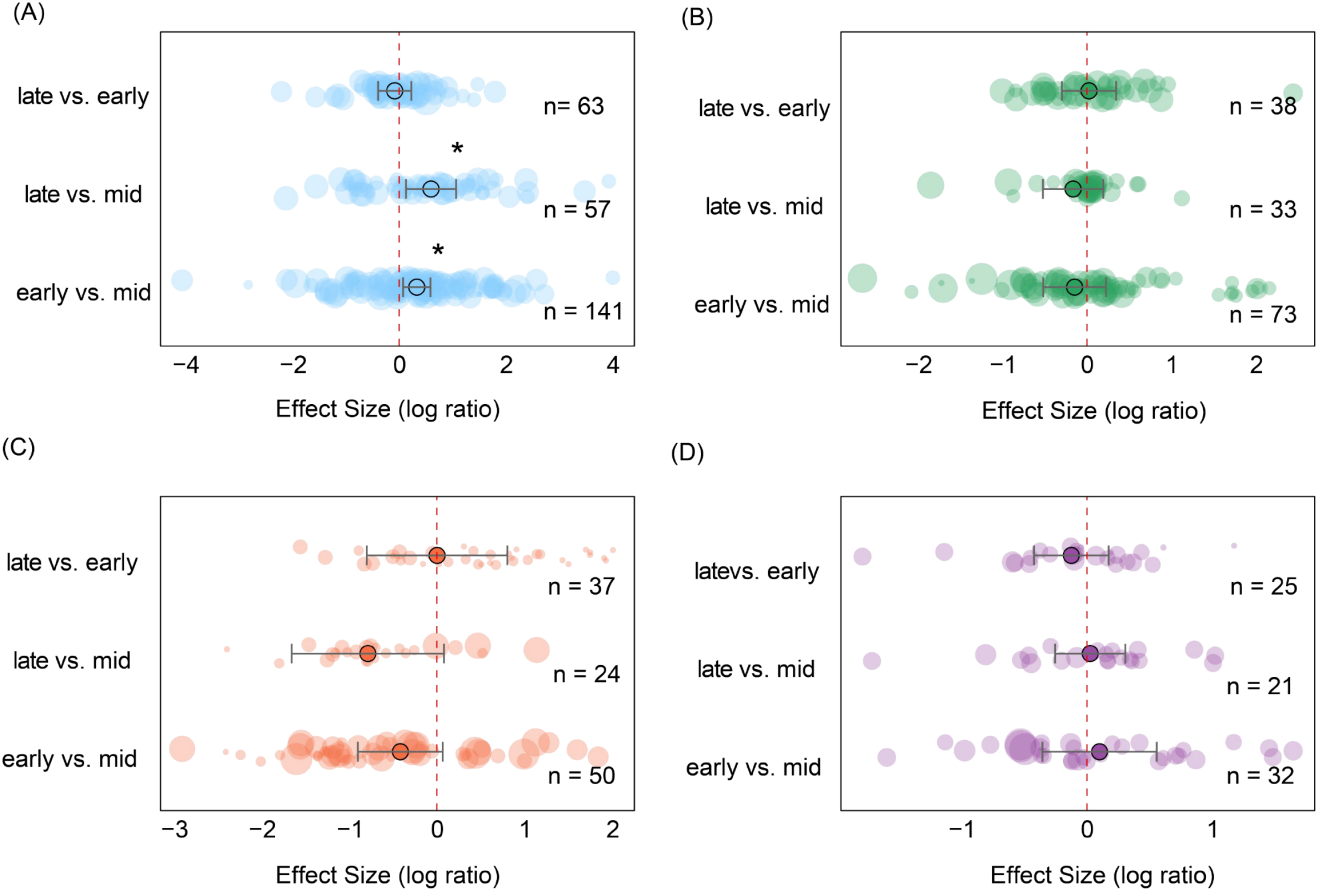
Changes in soil seed bank seed density across ecosystems based on pairwise comparisons between growing-season stages. **(A)** Grassland; **(B)** Forest; **(C)** Shrubland; **(D)** Farmland. Each point represents the effect size between two growing-season stages. Error bars denote 95% confidence intervals. Circle size corresponds to sample size in the respective comparison, with larger circles indicating greater sample numbers, and *n* represents the number of samples. Asterisks indicate statistical significance levels (*P < 0.05).

During the three growth stages, soil seed bank densities exhibited significant differences across the four ecosystem types (**Figure 7**). In grassland ecosystems, seed bank density during the mid-growing season was significantly lower than early-growing season (es = 0.32, CI = 0.07 to 0.58, *P* < 0.05) and late-growing season (es = 0.59, CI = 0.12 to 1.06; *P* < 0.05), while no significant differences were observed between the early-growing season and late-growing season (**Figure 7A**). This likely reflects the rapid germination and growth dynamics of grassland plants. In contrast, soil seed bank densities in forest, shrubs, and cropland ecosystems showed no significant differences across the three growth stages (**Figures 7B–D**). Overall, seasonal dynamics across ecosystems reflect differences in plant functional group life history strategies and distinct regulatory mechanisms governing soil seed banks across ecosystem types.

**Figure 8:**
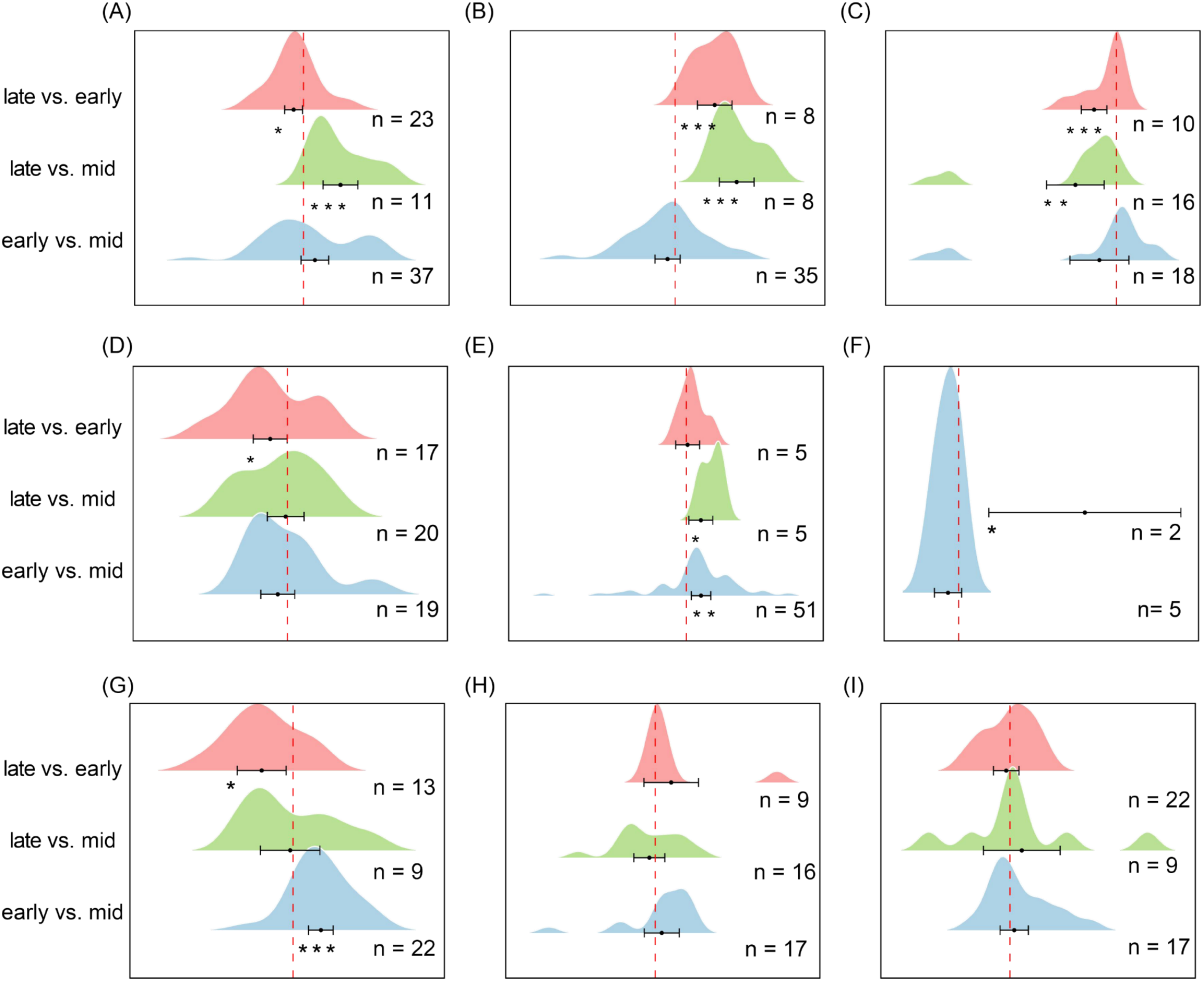
Pairwise comparisons of seed richness in soil seed banks across different ecosystems during successive stages of the growing season. **(A)** Alpine Meadow, **(B)** Temperate Deciduous Forest, **(C)** Tropical/Subtropical Forest, **(D)** Temperate Shrubland, **(E)** Temperate Steppe Grassland, **(F)** Boreal Fores, **(G)** Cropland, **(H)** Savannah, **(I)** Mediterranean Shrubland. Each ridge plot shows the distribution of effect sizes (log response ratios) across studies for three pairwise comparisons: early- vs. mid-season, late- vs. mid-season, and late- vs. early-season. Peaks indicate where most studies’ effect sizes are concentrated. Black dots represent the mean effect size for each comparison, with horizontal black lines indicating 95% confidence intervals. Sample sizes (n) are shown for each comparison, and asterisks (*) denote statistically significant differences (*P* < 0.05). This figure reflects species composition (richness), complementing **Figure 9**, which reflects seed abundance (density). ** P < 0.01, *** P < 0.001, indicating significant differences between treatments or groups.

Soil seed bank species richness exhibited significant seasonal variation across ecosystems, with distinct patterns among vegetation types. In alpine meadows (**Figure 8A**), richness during the late stage of the growing season was significantly higher than during the middle stage (es = 0.39, CI = 0.21 to 0.58, *P* < 0.001) but lower than during the early stage (es = -0.10, CI = -0.20 to 0.009, *P* < 0.05), indicating a temporary mid-season trough in seed bank richness. In temperate deciduous forests (**Figure 8B**), richness during the late stage was significantly higher than both the middle stage (es = 0.90, CI = 0.65 to 1.15, *P* < 0.001) and the early stage (es = 0.58, CI = 0.33 to 0.83, *P* < 0.001), suggesting a pronounced seed accumulation toward the end of the growing season. In contrast, tropical and subtropical forests (**Figure 8C**) exhibited the lowest richness during the late stage, which was significantly lower than both the middle stage (es = -0.50, CI = -0.85 to -0.15, *P* < 0.01) and the early stage (es = -0.27, CI = -0.43 to -0.11, *P* < 0.001), indicating substantial depletion of the seed bank by the end of the growing season.

In temperate shrublands (**Figure 8D**), richness during the early stage was significantly higher than during the late stage (es = -0.18, CI = -0.36 to -0.003, *P* < 0.05), suggesting seasonal loss of soil seed reserves. Temperate grasslands (**Figure 8E**) displayed a progressive decline, with richness significantly lower during the middle stage compared with the early stage (es = 0.185, CI = 0.063 to 0.307, *P* < 0.01) and further reduced during the late stage compared with the middle stage (es = 0.183, CI = 0.034 to 0.333, *P* < 0.05). In contrast, savannas and Mediterranean shrublands (**Figures 8H, I**) exhibited no significant differences among the three stages of the growing season (*P* > 0.05), maintaining relatively stable seed bank richness year-round. Together, these results highlight ecosystem-specific seasonal dynamics, including a mid-season trough in alpine meadows, late-season accumulation in temperate deciduous forests, late-season depletion in tropical forests, and progressive seasonal declines in temperate grasslands and shrublands, reflecting differences in seed input, germination phenology, and soil seed storage strategies.

**Figure 9:**
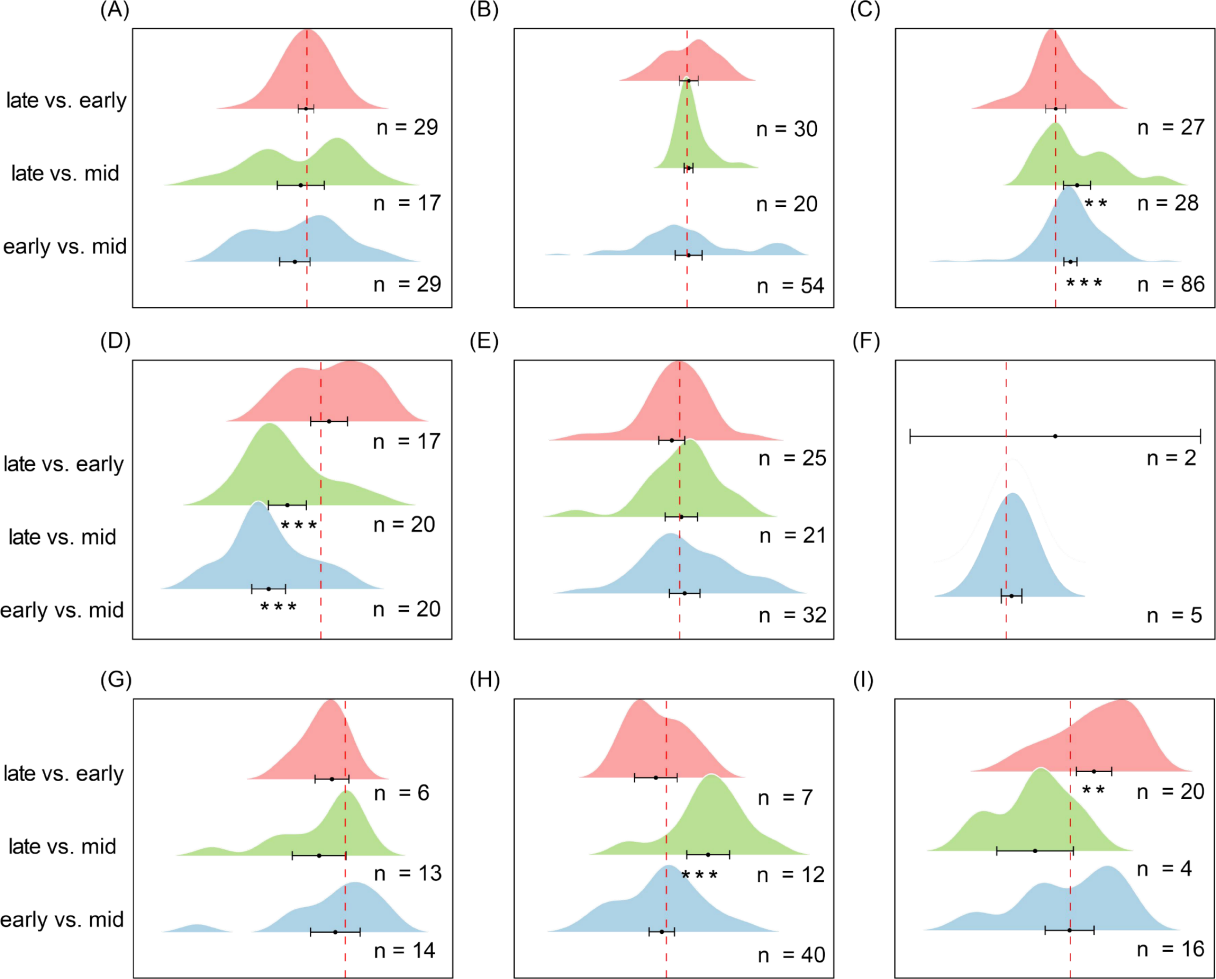
Changes in soil seed bank density across different ecosystem subtypes early-growing seasons, mid-growing seasons, and late-growing seasons. **(A)** Alpine Meadow, **(B)** Temperate Deciduous Forest, **(C)** Temperate Steppe Grassland, **(D)** Temperate Shrubland, **(E)** Cropland, **(F)** Boreal Fores, **(G)** Tropical/Subtropical Forest, **(H)** Savannah, **(I)** Mediterranean Shrubland. Each ridge plot shows the distribution of effect sizes (log response ratios) across studies for three pairwise comparisons: early- vs. mid-season, late- vs. mid-season, and late- vs. early-season. Peaks indicate where most studies’ effect sizes are concentrated. Black dots represent the mean effect size for each comparison, with horizontal black lines indicating 95% confidence intervals. Sample sizes (n) are shown for each comparison, and asterisks (*) denote statistically significant differences (*P* < 0.05). This figure depicts seed abundance (density), complementing **Figure 8**, which reflects species composition (richness). ** P < 0.01, *** P < 0.001, indicating significant differences between treatments or groups.

In temperate steppe grasslands (**Figure 9C**), seed density during the mid-growing season was significantly lower than both the pre-growing season (es = 0.530, CI = 0.30 to 0.77, *P* < 0.001) and the post-growing season (es = 0.768, CI = 0.28 to 1.26, *P* < 0.01). In contrast, in temperate shrublands (**Figure 9D**), seed density was significantly lower in the pre-growing season (es = -0.929, CI = -1.23 to -0.63, *P* < 0.001) and post-growing season (es = -0.595, CI = -0.93 to -0.26, *P* < 0.001) compared with the mid-growing season, indicating a density peak in mid-season. In savannas (**Figure 9H**), seed density in the post-growing season was significantly higher than in the mid-growing season (es = 0.932, CI = 0.45 to 1.41, *P* < 0.001), whereas in Mediterranean shrublands (**Figure 9I**), post-growing season seed density was significantly higher than pre-growing season density (es = 0.657, CI = 0.17 to 1.15, *P* < 0.01). In other ecosystems, seed density exhibited no significant seasonal variation, suggesting a relatively stable seasonal pattern.

## 4 Discussion

### 4.1 Richness and density of soil seed banks across growing season stages

Our findings indicate that soil seed banks exhibit a degree of seasonal fluctuation during the growing season: seed density is relatively high at the season’s onset, generally declines during the middle phase, and subsequently recovers to varying extents in the later stages. Although this trend did not reach statistical significance in some ecosystems, the overall pattern reflects the combined seasonal effects of processes such as germination, dispersal, mortality, and environmental selection. The elevated seed abundance observed at the onset of the growing season may stem from the combined contribution of persistent seeds carried over from the previous season and short-lived seeds released following early-season rainfall. This phenomenon exemplifies the soil seed bank’s capacity to serve as an “ecological buffer” by rapidly responding to environmental fluctuations (Ma et al., 2013b). As hydrothermal conditions improve, numerous species—particularly small-seeded, highly reproductive annual herbaceous plants and legumes—undergo synchronized germination during the mid-growing season (Del Vecchio et al., 2021). This seasonal synchronized germination reduces detectable seed numbers in the soil, leading to a widespread decline in mid-season seed density. Concurrently, stresses such as drought, high temperatures, predation, and pathogens may further accelerate seed consumption (Lennon et al., 2021). This seasonal rhythm, characterized by ‘early accumulation and mid-season depletion’, is closely linked to aboveground phenological changes: plants enter a rapid growth phase during the mid-season, while in the late season, the emergence of flowering and fruiting replenishes the soil seed bank with a new batch of seeds, partially restoring density (Quintana-Ascencio et al., 2019).

Differences in responses among species or functional groups to seasonal climatic drivers—such as early-season precipitation peaks and summer heatwaves—may further influence the rate and composition of seed bank replenishment across growth stages. For instance, woody plants exhibiting thick seed coats or deep dormancy often maintain dormancy during unfavorable seasons, thereby enhancing the contribution of persistent seed banks to interannual stability (Gioria et al., 2020). Consequently, transient seed banks more readily reflect seasonal changes in water and heat, whilst persistent seed banks maintain long-term population continuity through gradual renewal (Walck et al., 2005; DeMalach et al., 2021). Although seed density exhibits seasonal fluctuations, species richness within seed banks remains relatively stable throughout individual growing seasons. This may be attributed to species composition being governed by life history traits, functional group strategies, and competitive relationships, which change at a pace significantly slower than the seasonal variations in seed abundance (Yang et al., 2021). Furthermore, the asynchronous germination strategies of different species may also exert a buffering effect: some species tend to germinate early in the season, while others reach their germination peak later, thereby maintaining relatively stable overall species richness throughout seasonal variations (Wagg et al., 2022).

The seasonal dynamics of soil seed banks result from the combined effects of multiple ecological drivers: climatic seasonal variations determine the temporal windows for germination and replenishment, species life history strategies modulate their response intensity to resource pulses and stress events, while the complementarity between ephemeral and persistent seed banks enables ecosystems to respond rapidly to short-term hydrothermal fluctuations while maintaining long-term community continuity (Shen et al., 2007; Footitt et al., 2014; Johnson et al., 2022).

### 4.2 Changes in plant functional groups in the soil seed bank across growth stages

Different plant functional groups exhibit seasonally variable dynamics that are neither fully synchronized nor uniformly intense throughout the growing season. This characteristic reflects how life history strategies, trait combinations, and climatic seasonality jointly drive soil seed bank dynamics (Abd-ElGawad et al., 2025; Zhang et al., 2021). These variations mean seed banks are not solely influenced by quantitative changes but also exhibit multidimensional regulation in both composition and temporal structure.

Annual herbaceous plants and legumes typically exhibit higher activity and renewal rates during the early growing season. This trend is closely associated with their characteristic traits, including high seed production, small seed size, shorter dormancy periods, and greater tolerance to fluctuations in temperature and precipitation (Wyse and Dickie, 2018). In temperate arid regions where spring precipitation predominates, these rapid life history strategies enable them to exploit the brief water availability window in early seasons, facilitating rapid germination and regeneration. Consequently, seasonal fluctuations in the seed bank of such species tend to be more pronounced, with greater susceptibility to short-term variations as the season progresses. In contrast, woody plants such as vines and shrubs often possess larger seed sizes and more complex dormancy mechanisms, with germination frequently constrained by specific thermohygric conditions or low-temperature stratification (Ghasempour et al., 2022; Pritchard et al., 2022). This results in a slower or delayed renewal rhythm within their soil seed banks during the growing season. Although seed density in these species exhibits minimal short-term fluctuations, their response to climatic events—such as late-summer precipitation or autumn cold spells—may be more concentrated and exhibit a ‘triggered’ characteristic (Xin et al., 2021). Consequently, while their seasonal signals within the seed bank are weaker, they can play a crucial role at pivotal junctures.

The aforementioned differences indicate that life history strategies shape the degree of synchrony between aboveground phenology and belowground seed banks. In systems dominated by annual herbaceous plants, rapid germination and fruiting cause seed banks to more closely track seasonal precipitation and temperature rhythms, exhibiting relatively more pronounced seasonal trends (Woods et al., 2014; Liu et al., 2022). Conversely, in systems with greater woody species abundance, prolonged dormancy periods and asynchronous maturation generate buffering effects, moderating seasonal fluctuations (Abella and Springer, 2012). This phenomenon aligns with the ‘store-and-release’ regeneration strategy observed in arid environments (Ellner and Schreiber, 2012). Moreover, human activities may also influence seasonal dynamics among functional groups. For instance, in agroecosystems, management practices such as tillage, sowing, and harvesting establish temporal rhythms distinct from natural climatic patterns, often resulting in more concentrated and transient herbaceous community renewal (Van Daele et al., 2024). This anthropogenic seasonality may induce more rapid and unstable patterns of change in seed banks, contrasting with natural systems dominated by perennial plants (Buhler et al., 2001).

The seasonal variation in plant functional groups reflects the coupling between climatic rhythms, life history traits, and ecological processes. The asynchronous responses of different functional groups not only determine the seasonal structure of the soil seed bank but also influence the ecosystem’s resilience to disturbance or climatic fluctuations.

### 4.3 Seasonal dynamics of soil seed banks driven by ecosystem types

Seasonal variations in soil seed banks across different ecosystem types exhibit marked differences in magnitude and rhythmicity, primarily influenced by the combined effects of dominant life forms, seed traits, and fluctuations in water and thermal conditions. In ecosystems dominated by herbaceous plants—such as dry grasslands, agricultural fields, and alpine meadows—annual and short-lived species often play a more prominent role in seasonal dynamics. Characterized by small seed size, weak dormancy, and short regeneration cycles (Lyu et al., 2018), these species readily respond to precipitation pulses, early-season moisture conditions, and rising temperatures by rapid germination. Consequently, soil seed banks in these systems frequently exhibit a seasonal pattern of ‘relatively rapid depletion followed by replenishment’. This entails a noticeable decline in seed abundance during mid-growing season under favorable thermohygric conditions, subsequently replenished during the species’ fruiting period (Wang et al., 2009). This pattern aligns closely with the phenological processes of aboveground vegetation, reflecting the heightened sensitivity of herbaceous-dominated systems to short-term environmental fluctuations. In contrast, ecosystems dominated by perennial woody plants—such as forests, scrublands, Mediterranean maquis, and savannahs—tend to exhibit more moderate seasonal variations. Woody plant seeds typically possess thicker seed coats or stronger dormancy requirements, with germination often dependent on cold stratification, photoperiodic changes, or specific thermohygric conditions (Cheng et al., 2022). These characteristics result in longer soil residence times for woody seeds and a more dispersed germination window. Furthermore, the widespread occurrence of asynchronous fruiting and dispersed seed dispersal rhythms in woody plants provides a relatively stable seed input, thereby partially buffering seasonal fluctuations (Kiss et al., 2018). Due to slower regeneration rates, seed bank dynamics in such systems are more influenced by medium-to-long-term climatic processes than by short-term seasonal signals.

Disturbances and human activities further complicate these ecological patterns. Within agricultural ecosystems, tillage, sowing, and harvesting establish pronounced and regular anthropogenic seasonality, often synchronizing seed bank dynamics more closely with management rhythms than with natural climatic variations (Ortiz-Bobea et al., 2020). This facilitates the dominance of annual herbaceous species and may amplify the short-term amplitude of seasonal fluctuations. In contrast, tropical and subtropical forests frequently exhibit temporal mismatches between fruit ripening and optimal germination conditions. This asynchrony between input and establishment may constrain the formation of persistent seed banks, resulting in smoother or delayed seasonal dynamics (Chalermsri et al., 2020).

Ecosystems dominated by herbaceous plants rely more heavily on short-term seed supply and exhibit more pronounced seasonal patterns, whereas woody-dominated systems predominantly depend on long-term storage and gradual renewal to maintain greater temporal stability. These patterns are not mutually exclusive but reflect the balance different communities strike between short-term responsiveness and long-term stability. This perspective highlights the dual role of soil seed banks: supporting seasonal vegetation renewal at short timescales while maintaining community persistence and plant diversity at longer timescales (Gioria et al., 2021; Mašková and Poschlod, 2022).

## 5 Conclusion

This study systematically quantified the annual dynamics of soil seed banks globally, revealing significant seasonal variations across certain ecosystems and plant functional groups. Grassland ecosystems exhibited the most pronounced changes, indicating that soil seed banks are highly sensitive to environmental conditions and community structure. Differences in seed production and consumption timing among functional groups suggest that seed bank fluctuations are primarily influenced by the plant growing season and ecosystem characteristics. This study refines our understanding of soil seed bank dynamics, emphasizing that seasonal variation should be considered when assessing community renewal and composition.

## Data Availability

The original contributions presented in the study are included in the article/**Supplementary Material**, further inquiries can be directed to the corresponding author/s.
