# Supplementary material for: Global patterns in soil seed bank seasonality: a meta-analysis of ecosystem and functional group dynamics
Source: Front Plant Sci. 2025 Dec 17;16:1725507. doi: 10.3389/fpls.2025.1725507 (PMC12753970; doi:10.3389/fpls.2025.1725507)
Supplement: Supplementary file 1 [file DataSheet1.docx]

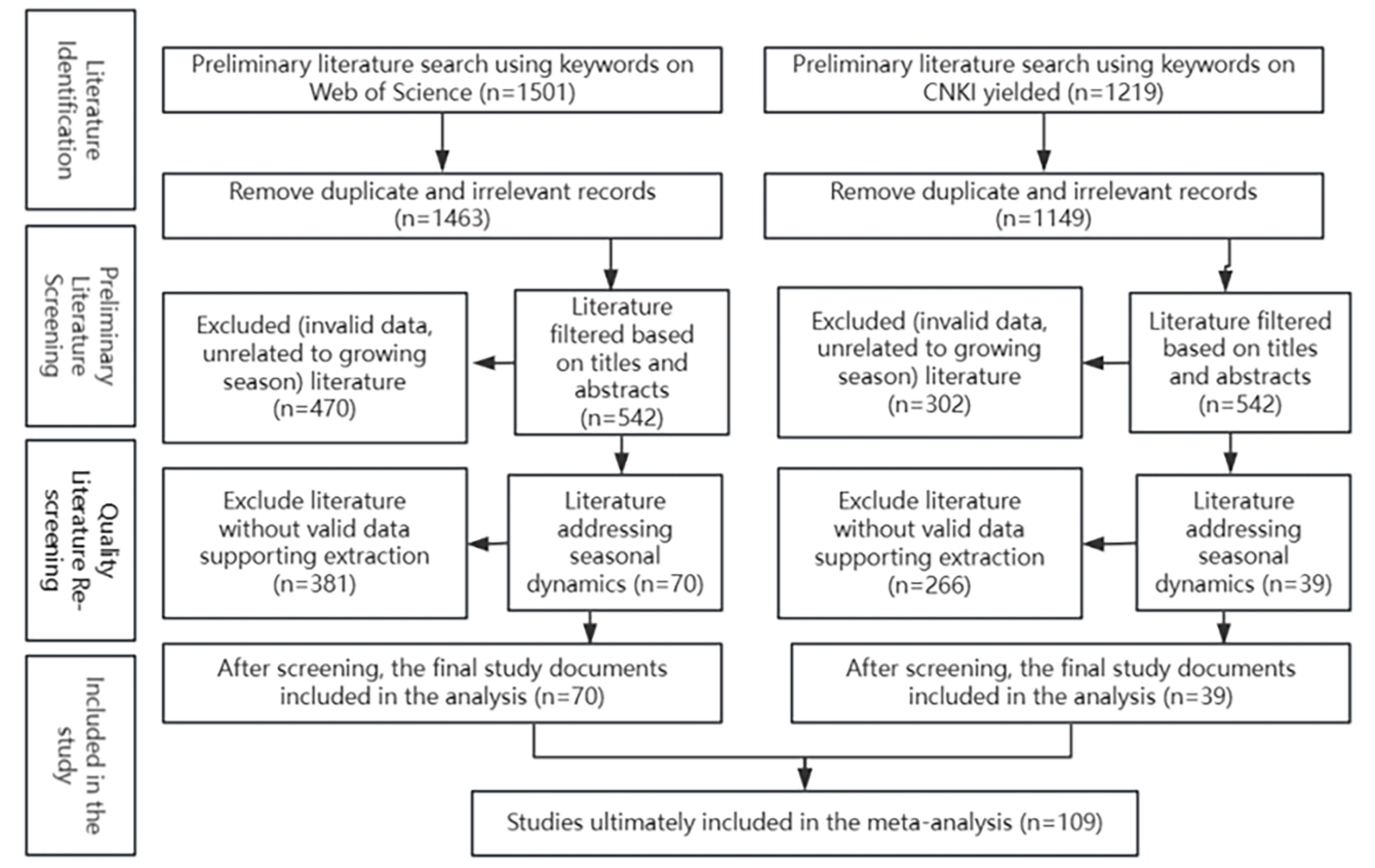


**Supplementary Figure S1:** Preferred Reporting Items for Systematic Reviews and Meta-Analyses Flow Diagram


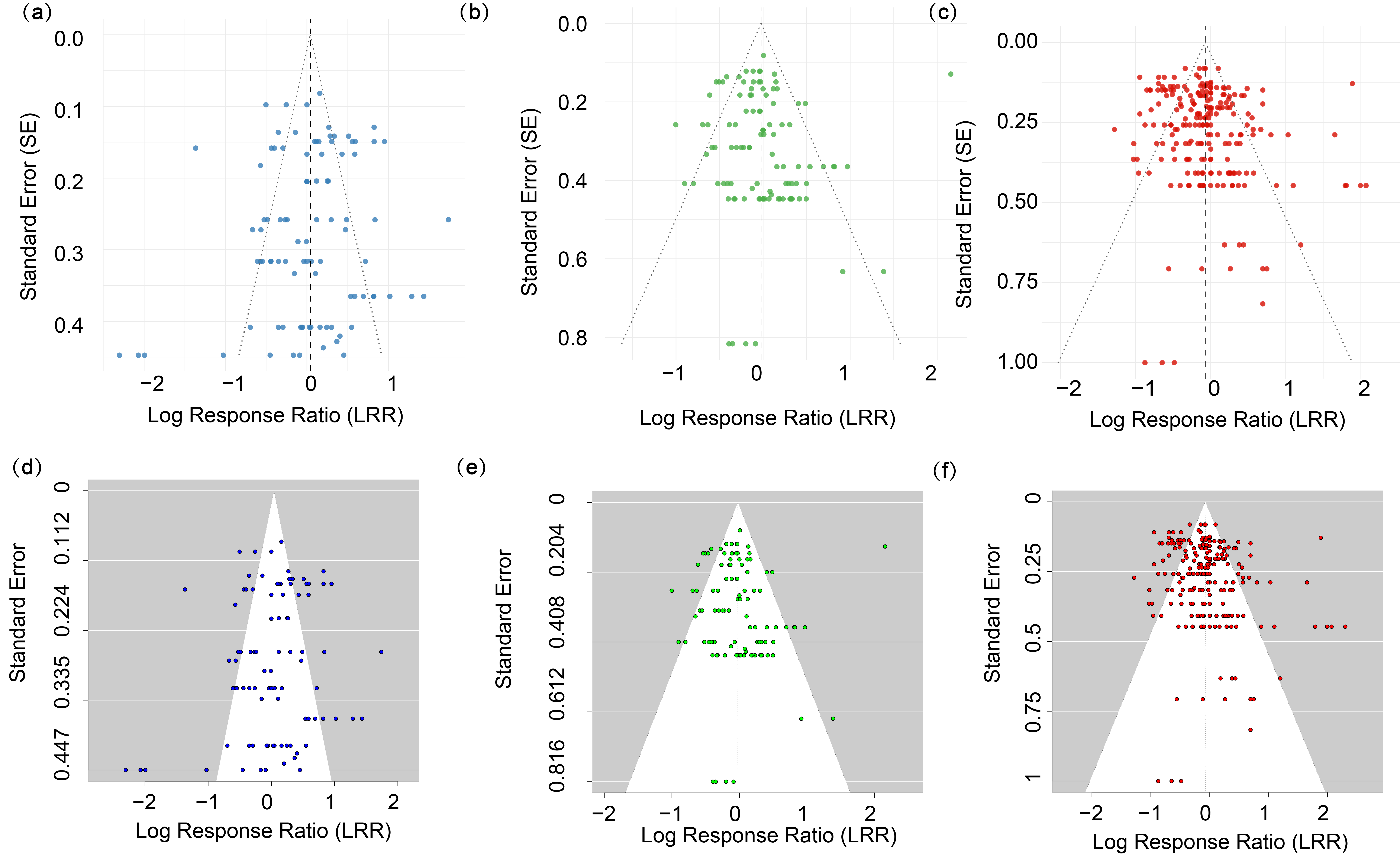


**Supplementary Figure S2:** Funnel plots and Trim and Fill–adjusted results for richness comparisons across growing season stages. Panels (a–c) show the original funnel plots for early- vs. mid-season, late- vs. mid-season, and late- vs. early-season comparisons, while (d–f) present the corresponding Trim and Fill–adjusted plots. Egger’s regression tests revealed significant publication bias for early- vs. mid-season (t = 2.51, *P* = 0.0130), but no bias for late- vs. mid-season (*P* = 0.6195) or late- vs. early-season (*P* = 0.9595). After Trim and Fill correction, the plots became more symmetrical, indicating limited publication bias and more reliable estimates of richness effect sizes (LRR).


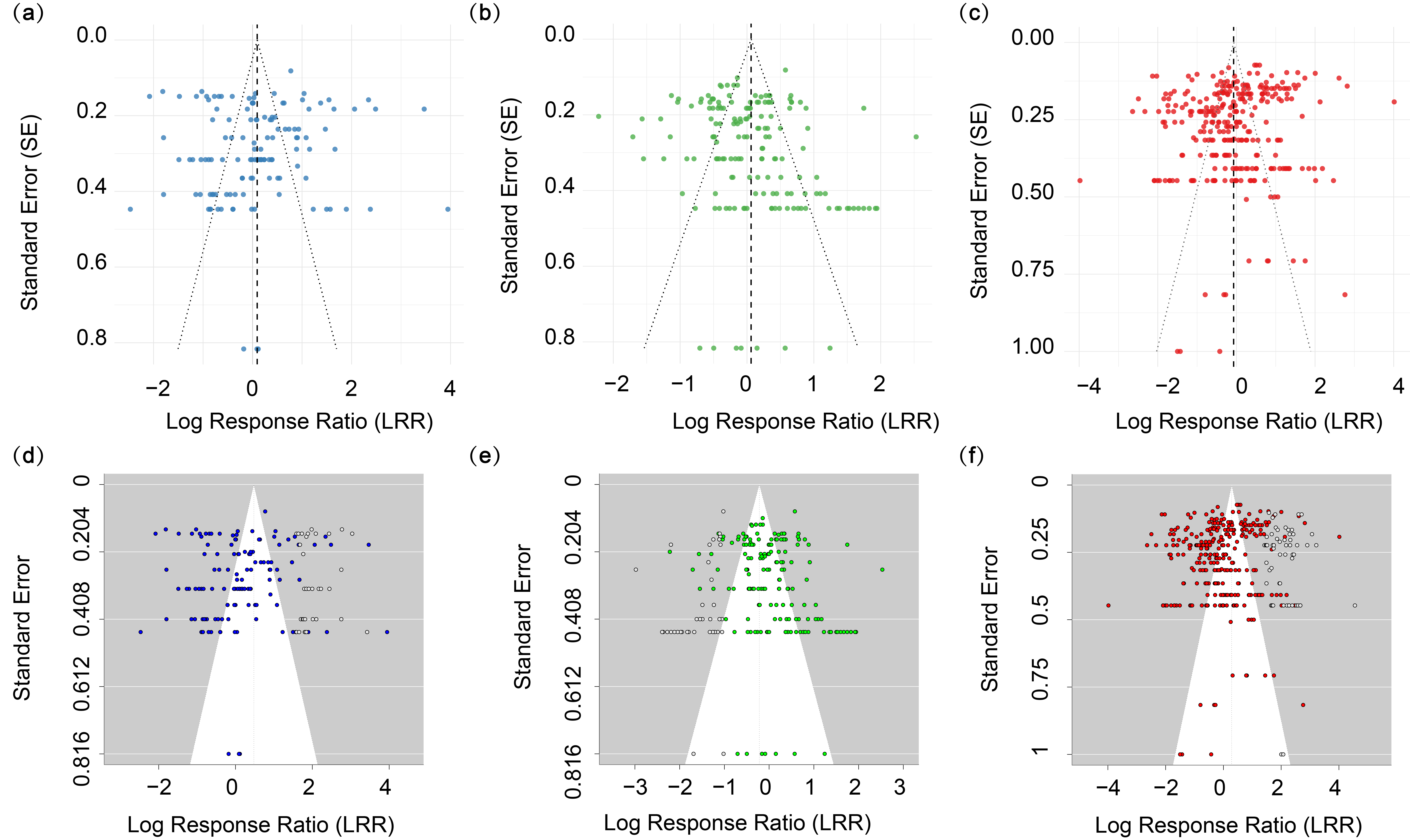


**Supplementary Figure S3:** Funnel plots and Trim and Fill–adjusted results for density comparisons across growing season stages. Panels (a–c) show the original funnel plots for early- vs. mid-season, late- vs. mid-season, and late- vs. early-season comparisons, while (d–f) present the corresponding Trim and Fill–adjusted plots. Egger’s regression tests indicated significant asymmetry for early- vs. mid-season (t = -2.96, *P*= 0.0033), no bias for late- vs. mid-season (*P* = 0.7979), and mild asymmetry for late- vs. early-season (t = 2.02, *P* = 0.0455). After Trim and Fill correction, the plots became more symmetrical, suggesting that the bias-corrected effect size (LRR) estimates for density are more robust.

Three-level meta-analysis of overall effects and heterogeneity components.


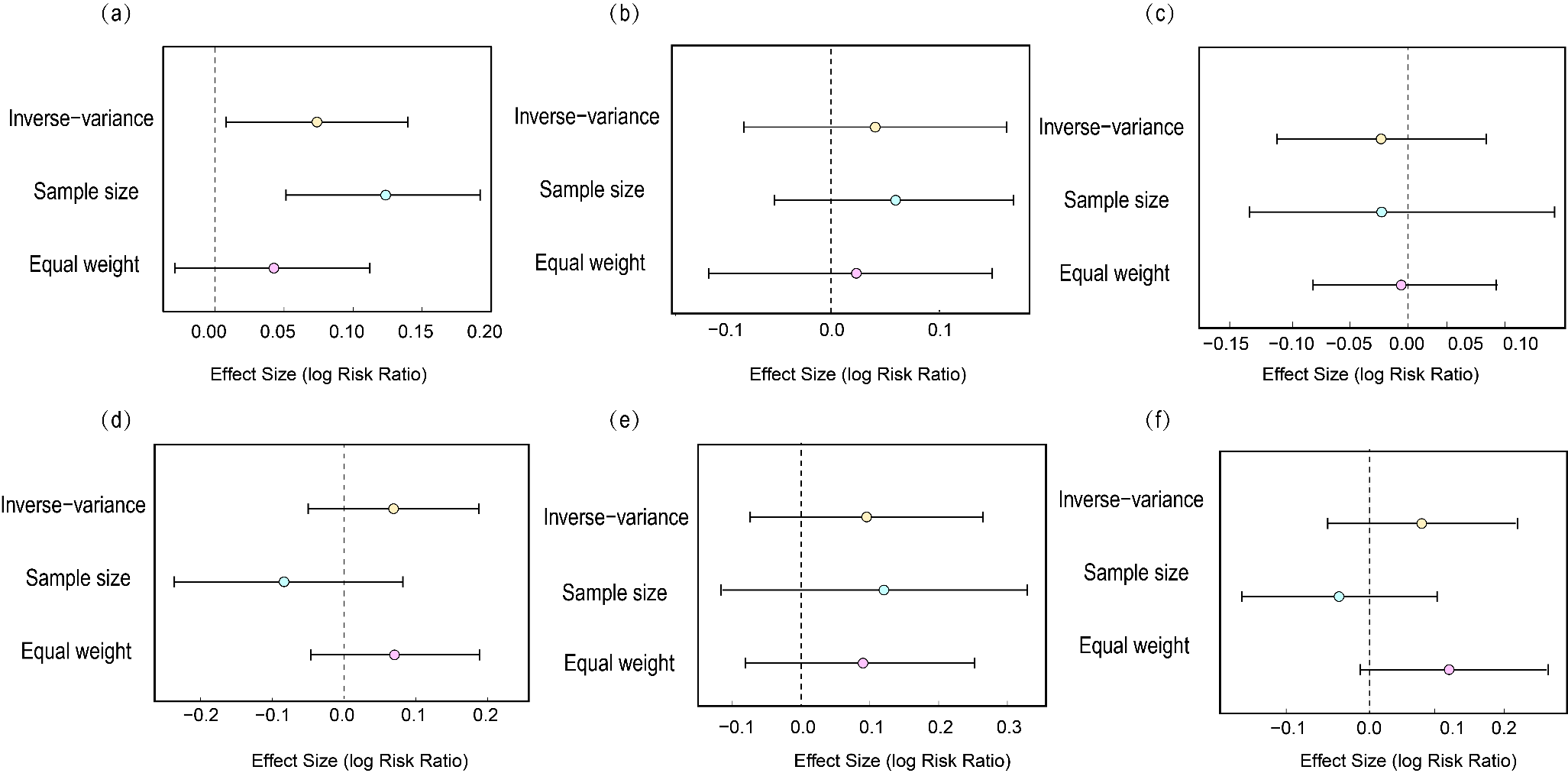


**Supplementary Figure S4:** Different weighting–scheme comparison plots. Panels (a) and (d) show the Early vs. Mid comparison, panels (b) and (e) show the Late vs. Mid comparison, and panels (c) and (f) show the Late vs. Early comparison. Panels (a–c) depict richness, while panels (d–f) depict density. Sensitivity analyses indicate that across all pairwise comparisons among the three stages, the meta-analysis results remain robust regardless of whether inverse-variance, sample-size, or equal-weighting schemes are applied.


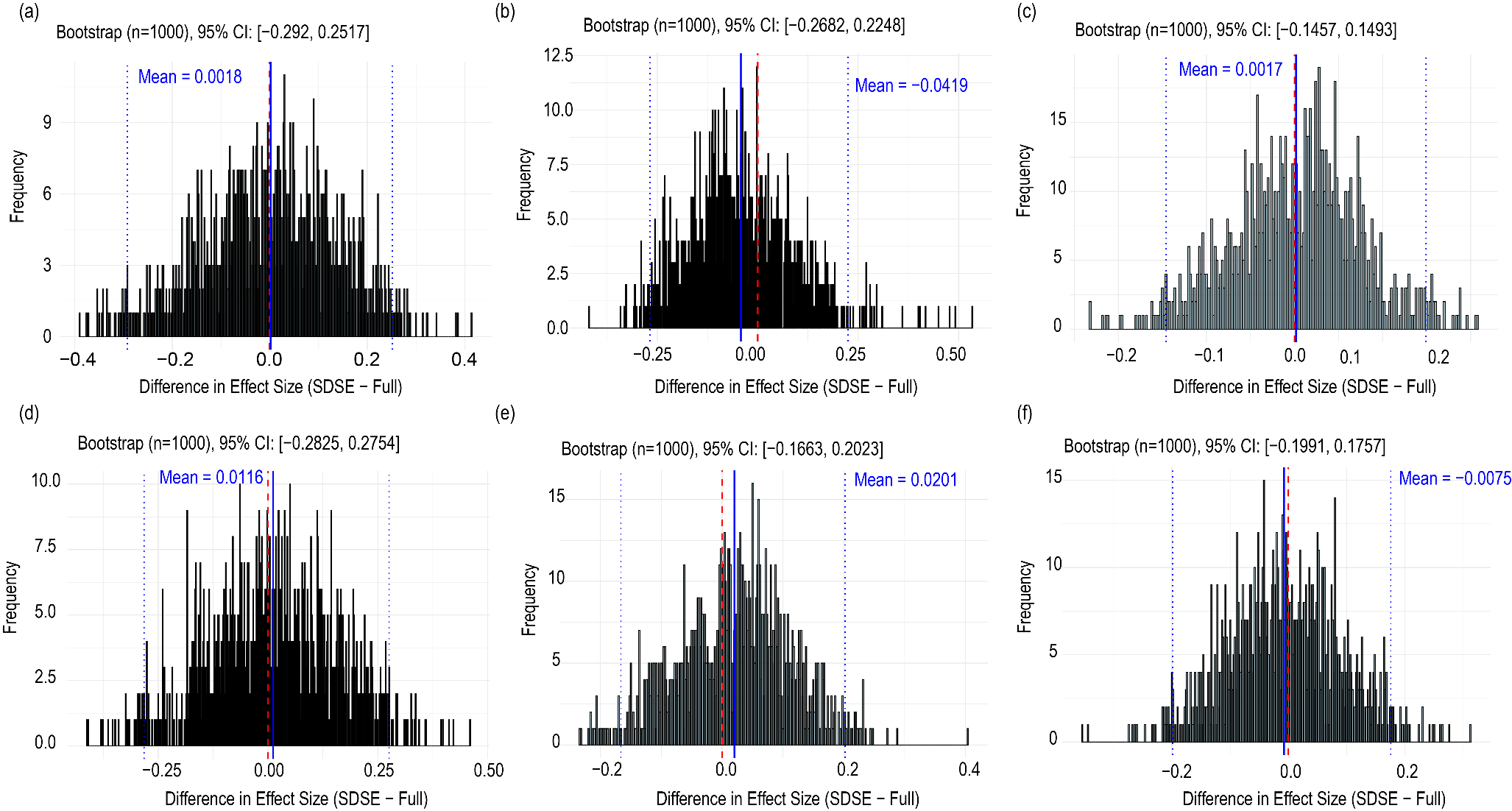


**Supplementary Figure S5:** Distribution of effect size differences between the SDSE subset analysis and the full dataset analysis based on 1000 bootstrap resamples. Panels (a) and (d) show the Late vs. Mid comparison, panels (b) and (e) show the Late vs. Early comparison, and panels (c) and (f) show the Early vs. Mid comparison. Panels (a–c) depict richness, while panels (d–f) depict density. For each bootstrap iteration, the full dataset and the SDSE subset were resampled with replacement and refitted using a random‐effects model. The histogram displays the resulting distribution of SDSE − Full effect size differences. The red dashed line denotes zero difference, and the blue vertical line indicates the bootstrap mean across resamples.


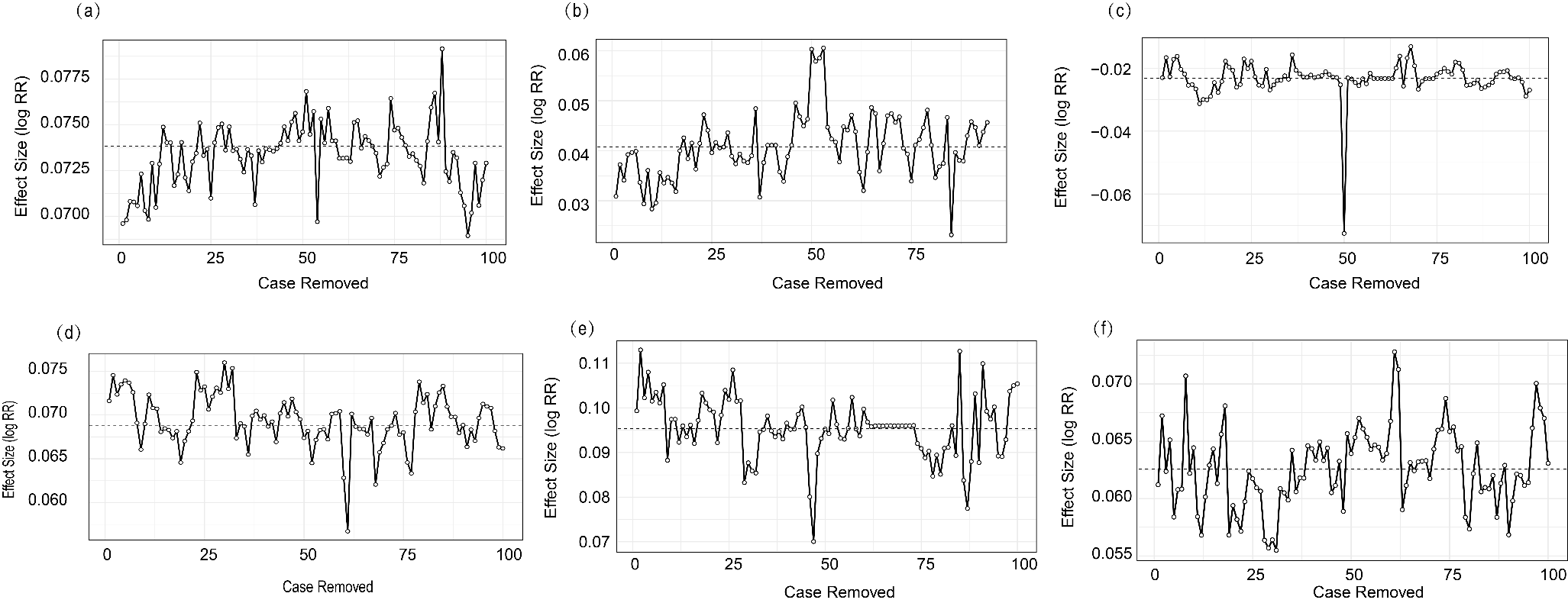


**Supplementary Figure 6:** Leave-one-out (LOO) sensitivity analysis plot. Panels (a) and (d) show the Early vs. Mid comparison, panels (b) and (e) show the Late vs. Mid comparison, and panels (c) and (f) show the Late vs. Early comparison. Panels (a–c) present results for richness, whereas panels (d–f) correspond to density. Although the leave-one-out procedure yielded small positive and negative shifts in the estimated effect sizes, these fluctuations were minor and did not alter the overall conclusions for the density comparisons across growing-season stages, demonstrating the robustness of the results.

| Comparison | Studies | Effects | τ² | I² | Q | df | p |
| --- | --- | --- | --- | --- | --- | --- | --- |
| Richness: early vs mid | 61 | 221 | 0.1801 | 62.1% | 1131.33 | 220 | <0.001 |
| Richness: late vs mid | 24 | 94 | 0.2834 | 75.7% | 581.23 | 93 | <0.001 |
| Richness: late vs early | 23 | 109 | 0.1679 | 55.6% | 467.11 | 108 | <0.001 |
| Density: early vs mid | 79 | 296 | 0.9778 | 90.3% | 8207.18 | 295 | <0.001 |
| Density: late vs mid | 33 | 135 | 0.9479 | 90.4% | 2768.82 | 134 | <0.001 |
| Density: late vs early | 35 | 163 | 0.4735 | 80.4% | 1373.98 | 162 | <0.001 |

**Supplementary** **Table S1:** Meta-analysis results comparing species richness and density among growing-season stages. Overall effects were weak, but heterogeneity among studies was significant.

| Comparison | Studies | Effects | τ² | I² | Q | df | p |
| --- | --- | --- | --- | --- | --- | --- | --- |
| Richness: T+P-II vs P | 71 | 71 | 0.1162 | 57.5% | 269.03 | 70 | <0.001 |
| Richness: T+P-I vs P | 28 | 28 | 0.0596 | 35.3% | 61.28 | 27 | <0.001 |
| Richness: T+P-I vs T+P-II | 28 | 28 | 0.0620 | 36.2% | 60.11 | 27 | <0.001 |
| Density: T+P-II vs P | 69 | 69 | 0.9725 | 89.3% | 1328.26 | 68 | <0.001 |
| Density: T+P-I vs P | 36 | 36 | 0.4331 | 74.6% | 231.45 | 35 | <0.001 |
| Density: T+P-I vs T+P-II | 42 | 42 | 0.5740 | 81.7% | 853.35 | 41 | <0.001 |

**Supplementary** **Table S2:** Comparison of species richness and seed density between temporary and permanent seed banks. Overall, temporary seed banks exhibit higher species richness and seed density than permanent seed banks, with seed density showing greater variability, suggesting higher sensitivity to seasonal dynamics and environmental fluctuations

| Group | Comparison | Studies | τ² | I² | Q | df |
| --- | --- | --- | --- | --- | --- | --- |
| Density | annual | Early vs Mid | 17 | 0.21 | 81.39 | 90.95 |
| Density | annual | Late vs Mid | 8 | 0.14 | 73.53 | 48.68 |
| Density | annual | Late vs Early | 9 | 0.04 | 42.57 | 13.85 |
| Density | cyearlyaceae | Late vs Early | 4 | 0.62 | 92.56 | 40.35 |
| Density | gramineae | Early vs Mid | 3 | 0.05 | 71.16 | 6.39 |
| Density | herb | Early vs Mid | 27 | 0.44 | 89.51 | 442.82 |
| Density | herb | Late vs Mid | 15 | 0.31 | 75.07 | 52.96 |
| Density | herb | Late vs Early | 12 | 0.32 | 77.93 | 44.51 |
| Density | legume | Early vs Mid | 2 | 0.64 | 95.82 | 23.92 |
| Density | liana | Early vs Mid | 5 | 7.17 | 99.37 | 765.87 |
| Density | earlyennial | Early vs Mid | 15 | 0.25 | 84.59 | 103.93 |
| Density | earlyennial | Late vs Mid | 8 | 0.11 | 69.03 | 37.85 |
| Density | earlyennial | Late vs Early | 9 | 0.28 | 85.55 | 137.26 |
| Density | shrub | Early vs Mid | 18 | 0.78 | 94.65 | 374.11 |
| Density | shrub | Late vs Mid | 11 | 1.41 | 95.94 | 461.17 |
| Density | shrub | Late vs Early | 8 | 1.30 | 96.46 | 323.93 |
| Density | tree | Early vs Mid | 1 | 0.00 | 0.00 | 0.00 |
| Density | woody | Early vs Mid | 6 | 0.67 | 90.96 | 57.44 |
| Richness | annual | Early vs Mid | 27 | 0.38 | 87.58 | 182.82 |
| Richness | annual | Late vs Mid | 2 | 0.00 | 0.00 | 0.02 |
| Richness | annual | Late vs Early | 3 | 0.00 | 0.00 | 0.54 |
| Richness | cyearlyaceae | Early vs Mid | 2 | 0.17 | 72.25 | 3.60 |
| Richness | cyearlyaceae | Late vs Early | 4 | 0.00 | 0.00 | 2.70 |
| Richness | gramineae | Early vs Mid | 14 | 0.53 | 90.33 | 151.62 |
| Richness | gramineae | Late vs Early | 1 | 0.00 | 0.00 | 0.00 |
| Richness | herb | Early vs Mid | 3 | 0.44 | 76.77 | 8.61 |
| Richness | herb | Late vs Mid | 3 | 0.60 | 81.87 | 11.03 |
| Richness | herb | Late vs Early | 5 | 0.16 | 54.43 | 8.78 |
| Richness | legume | Early vs Mid | 6 | 0.58 | 90.64 | 67.33 |
| Richness | legume | Late vs Early | 2 | 0.00 | 0.00 | 0.00 |
| Richness | liana | Early vs Mid | 7 | 0.82 | 92.25 | 146.69 |
| Richness | liana | Late vs Mid | 4 | 0.00 | 0.00 | 2.52 |
| Richness | liana | Late vs Early | 6 | 0.22 | 62.47 | 13.32 |
| Richness | earlyennial | Early vs Mid | 27 | 0.20 | 79.21 | 113.19 |
| Richness | earlyennial | Late vs Mid | 3 | 0.00 | 0.00 | 0.97 |
| Richness | earlyennial | Late vs Early | 5 | 0.03 | 26.44 | 6.75 |
| Richness | shrub | Early vs Mid | 16 | 0.27 | 82.99 | 78.15 |
| Richness | shrub | Late vs Mid | 9 | 0.09 | 52.56 | 18.86 |
| Richness | shrub | Late vs Early | 9 | 0.33 | 80.41 | 36.95 |
| Richness | tree | Early vs Mid | 11 | 0.00 | 0.00 | 5.77 |
| Richness | tree | Late vs Mid | 8 | 0.00 | 0.00 | 3.36 |
| Richness | tree | Late vs Early | 8 | 0.00 | 0.00 | 2.32 |
| Richness | woody | Early vs Mid | 9 | 0.00 | 0.00 | 2.07 |
| Richness | woody | Late vs Mid | 8 | 0.00 | 0.00 | 0.70 |
| Richness | woody | Late vs Early | 8 | 0.00 | 0.00 | 2.15 |

**Supplementary** **Table S3:** Meta-analysis results of seed bank species richness and density across growing-season stages (early, mid, and late) for different plant functional groups. Overall, changes in species richness and seed density across stages were generally small. Most functional groups exhibited high heterogeneity in density and moderate to high heterogeneity in richnes, indicating variability among studies. Tree and woody species showed low heterogeneity (I² = 0%), suggesting more consistent responses across studies.

| Group | Comparison | τ² | I² | Q | df |
| --- | --- | --- | --- | --- | --- |
| Richness-Grassland | Pre vs During | 0.206 | 63.0 | 682.17 | 98 |
| Richness-Forest | Pre vs During | 0.225 | 65.7 | 223.34 | 57 |
| Richness-Shrubland | Pre vs During | 0.124 | 56.8 | 129.15 | 41 |
| Richness-Cropland | Pre vs During | 0.036 | 34.4 | 32.61 | 21 |
| Richness-Grassland | Post vs During | 0.226 | 81.1 | 248.53 | 28 |
| Richness-Forest | Post vs During | 0.511 | 79.9 | 133.74 | 23 |
| Richness-Shrubland | Post vs During | 0.200 | 65.7 | 134.04 | 31 |
| Richness-Cropland | Post vs During | 0.237 | 79.0 | 32.17 | 8 |
| Richness-Grassland | Post vs Pre | 0.201 | 80.3 | 337.52 | 36 |
| Richness-Forest | Post vs Pre | 0.255 | 60.6 | 48.96 | 19 |
| Richness-Shrubland | Post vs Pre | 0.021 | 11.8 | 31.22 | 38 |
| Richness-Cropland | Post vs Pre | 0.115 | 31.7 | 25.63 | 12 |
| Density-Grassland | Pre vs During | 0.965 | 89.5 | 4279.53 | 140 |
| Density-Forest | Pre vs During | 0.729 | 85.2 | 1258.05 | 72 |
| Density-Shrubland | Pre vs During | 1.204 | 94.1 | 1619.36 | 49 |
| Density-Cropland | Pre vs During | 0.620 | 89.9 | 374.17 | 31 |
| Density-Grassland | Post vs During | 1.185 | 91.7 | 1969.18 | 56 |
| Density-Forest | Post vs During | 0.277 | 77.2 | 231.35 | 32 |
| Density-Shrubland | Post vs During | 1.029 | 89.0 | 226.06 | 23 |
| Density-Cropland | Post vs During | 0.351 | 81.7 | 127.55 | 20 |
| Density-Grassland | Post vs Pre | 0.517 | 84.9 | 693.28 | 62 |
| Density-Forest | Post vs Pre | 0.376 | 85.0 | 345.14 | 37 |
| Density-Shrubland | Post vs Pre | 0.950 | 85.0 | 220.41 | 36 |
| Density-Cropland | Post vs Pre | 0.227 | 56.9 | 85.79 | 24 |

**Supplementary** **Table S4:** Meta-analysis results of soil seed bank species richness and density across growing-season stages (early, mid, late) for different ecosystem types (Grassland, Forest, Shrubland, Cropland). Overall, changes in richness and density across stages were generally small. Species richness showed moderate to high heterogeneity, while seed density exhibited high heterogeneity (I² = 56.9–94.1%), indicating variability among studies. Grasslands and shrublands had the highest density heterogeneit, whereas richness heterogeneity in forests and croplands was relatively lowe, suggesting more consistent responses in these ecosystems.

| Group | Comparison | τ² | I² | Q | df |
| --- | --- | --- | --- | --- | --- |
| Density_Alpine Meadow | Post vs During | 0.933 | 96.7 | 451.00 | 16 |
| Density_Alpine Meadow | Post vs Pre | 0.145 | 83.3 | 170.00 | 28 |
| Density_Alpine Meadow | Pre vs During | 0.591 | 93.3 | 420.00 | 28 |
| Density_Boreal forest | Post vs Pre | 4.560 | 98.6 | 69.40 | 1 |
| Density_Boreal forest | Pre vs During | 0.038 | 65.3 | 11.50 | 4 |
| Density_Cropland | Post vs During | 0.351 | 83.4 | 127.55 | 20 |
| Density_Cropland | Post vs Pre | 0.216 | 72.5 | 85.79 | 24 |
| Density_Cropland | Pre vs During | 0.525 | 93.7 | 374.17 | 31 |
| Density_Mediterranean woodland | Post vs During | 1.050 | 90.0 | 24.90 | 3 |
| Density_Mediterranean woodland | Post vs Pre | 1.060 | 85.9 | 159.00 | 19 |
| Density_Mediterranean woodland | Pre vs During | 1.850 | 98.4 | 945.65 | 15 |
| Density_Savannah | Post vs During | 0.671 | 95.0 | 279.31 | 11 |
| Density_Savannah | Post vs Pre | 0.361 | 90.0 | 46.10 | 6 |
| Density_Savannah | Pre vs During | 0.784 | 96.7 | 1326.79 | 39 |
| Density_Temperate forest | Post vs During | 1.580 | 96.4 | 609.49 | 27 |
| Density_Temperate forest | Post vs Pre | 0.774 | 93.6 | 440.39 | 26 |
| Density_Temperate forest | Pre vs During | 1.140 | 96.7 | 2568.50 | 85 |
| Density_Temperate grassland | Post vs During | <0.001 | 0.0 | 13.90 | 19 |
| Density_Temperate grassland | Post vs Pre | 0.233 | 88.1 | 238.80 | 29 |
| Density_Temperate grassland | Pre vs During | 0.932 | 95.4 | 1086.83 | 53 |
| Density_Tropical forest | Post vs During | 0.474 | 85.7 | 186.67 | 19 |
| Density_Tropical forest | Post vs Pre | 0.331 | 70.3 | 53.50 | 16 |
| Density_Tropical forest | Pre vs During | 0.349 | 77.8 | 79.94 | 19 |
| Density_Tropical savanna | Post vs During | 0.359 | 86.2 | 137.39 | 12 |
| Density_Tropical savanna | Post vs Pre | 0.000 | 0.0 | 2.77 | 5 |
| Density_Tropical savanna | Pre vs During | 0.276 | 79.3 | 54.36 | 13 |
| Richness_Alpine Meadow | Post vs During | 0.070 | 73.2 | 38.60 | 10 |
| Richness_Alpine Meadow | Post vs Pre | 0.027 | 50.8 | 44.00 | 22 |
| Richness_Alpine Meadow | Pre vs During | 0.133 | 81.8 | 168.46 | 36 |
| Richness_Boreal forest | Post vs Pre | 0.000 | 0.0 | 0.28 | 1 |
| Richness_Boreal forest | Pre vs During | 0.000 | 0.0 | 2.24 | 4 |
| Richness_Cropland | Post vs During | 0.176 | 74.4 | 32.17 | 8 |
| Richness_Cropland | Post vs Pre | 0.115 | 57.1 | 25.63 | 12 |
| Richness_Cropland | Pre vs During | 0.036 | 37.3 | 32.61 | 21 |
| Richness_Mediterranean woodland | Post vs During | 0.470 | 87.4 | 55.70 | 8 |
| Richness_Mediterranean woodland | Post vs Pre | 0.000 | 0.0 | 13.70 | 21 |
| Richness_Mediterranean woodland | Pre vs During | 0.082 | 63.2 | 41.20 | 16 |
| Richness_Savannah | Post vs During | 0.285 | 93.1 | 196.28 | 15 |
| Richness_Savannah | Post vs Pre | 0.507 | 94.3 | 214.86 | 8 |
| Richness_Savannah | Pre vs During | 0.392 | 93.7 | 324.83 | 16 |
| Richness_Temperate forest | Post vs During | 0.000 | 0.0 | 0.92 | 4 |
| Richness_Temperate forest | Post vs Pre | 0.000 | 0.0 | 0.64 | 4 |
| Richness_Temperate forest | Pre vs During | 0.126 | 76.9 | 202.65 | 50 |
| Richness_Temperate grassland | Post vs During | 0.000 | 0.0 | 5.51 | 7 |
| Richness_Temperate grassland | Post vs Pre | 0.000 | 0.0 | 4.54 | 7 |
| Richness_Temperate grassland | Pre vs During | 0.205 | 77.2 | 129.76 | 34 |
| Richness_Tropical forest | Post vs During | 0.084 | 51.5 | 37.70 | 19 |
| Richness_Tropical forest | Post vs Pre | <0.001 | 0.0 | 16.30 | 16 |
| Richness_Tropical forest | Pre vs During | 0.042 | 29.1 | 24.40 | 18 |
| Richness_Tropical savanna | Post vs During | 0.394 | 83.2 | 55.90 | 15 |
| Richness_Tropical savanna | Post vs Pre | 0.000 | 0.0 | 5.94 | 9 |
| Richness_Tropical savanna | Pre vs During | 0.517 | 92.8 | 85.04 | 17 |

**Supplementary** **Table S5:** Meta-analysis results of soil seed bank species richness and density across growing-season stage for different ecosystem types (Alpine Meadow, Boreal Forest, Cropland, Mediterranea Woodland, Savannah, Temperate Forest/Grassland, Tropical Forest/Savanna). Overall, average changes in richness and density across stages were generally small. Species richness showed moderate to high heterogeneity, while seed density exhibited high heterogeneit, indicating some variability in responses among studies. Seed density heterogeneity was highest in Alpine Meadows, Tropical Savannas, and Temperate Forest, whereas richness heterogeneity in some forests and croplands was relatively lower, suggesting more consistent responses in these ecosystems.
